# TEM analysis of iron nanoparticle-loaded endophytic bacteria mitigating salinity-induced root stress effects in maize

**DOI:** 10.1186/s12934-026-03106-7

**Published:** 2026-09-05

**Authors:** Amany M. Reyad, Yasmeen M. Fathy, Aya M. Rabie, Tharwat E. E. Radwan, Karima Ali Sayed

**Affiliations:** Botany Department, Faculty of Science, Fayoum University, Fayoum, Egypt

**Keywords:** Nano-bio hybrid, Endophytic *Bacillus*, Iron nanoparticles (FeNPs), Salinity stress tolerance

## Abstract

Salinity stress is a major abiotic constraint limiting crop productivity through osmotic imbalance, ionic toxicity, oxidative damage, and structural disruption. Here, we report a novel nano-enabled microbial cell factory system based on iron nanoparticle (FeNP)-loaded plant growth-promoting endophytic bacteria (PGPEB) isolated from *Zea mays* roots and identified as *Bacillus* sp. (16S rRNA accession MZ700077.1; 99.41% similarity). This living nano-bio hybrid was designed to integrate microbial plant growth-promoting functions with nanoparticle-mediated micronutrient delivery for enhanced salinity stress tolerance. Transmission electron microscopy confirmed successful formation of the microbial cell factory, revealing rod-shaped bacterial cells with intact flagella and uniform spherical FeNPs (~ 29.9 nm) strongly associated with the bacterial surface, indicating stable nano-bio interfacing without compromising cellular integrity. The engineered FeNP-PGPEB system significantly improved maize seed germination, root and shoot growth, and overall seedling vigor under moderate to severe salinity (200 mM NaCl), outperforming either PGPEB or FeNPs alone and demonstrating clear synergistic effects. Physiological and biochemical analyses revealed that the nano-bio formulation enhanced antioxidant defense systems (SOD, CAT, POD), increased proline accumulation, and optimized phytohormonal balance by elevating IAA and moderating ABA levels. In addition, lipid peroxidation (MDA) was reduced, while relative water content was maintained, indicating improved redox homeostasis and osmotic regulation. Ultrastructural and anatomical observations further confirmed reduced plasmolysis, improved membrane integrity, and enhanced vascular and root tissue organization under salt stress. Multivariate analyses (PCA and clustering) clearly distinguished treatments, with FeNP–PGPEB strongly associated with stress resilience traits and improved physiological stability. Overall, this study establishes a novel microbial cell factory platform, where endophytic *Bacillus* acts as a living nanocarrier for iron delivery, integrating microbial biotechnology and nanotechnology into a unified strategy for enhancing crop tolerance to salinity stress and advancing sustainable agricultural systems.

## Introduction

Soil salinity is one of the most destructive abiotic constraints to global agricultural productivity, particularly in arid and semi-arid regions where high evapotranspiration, limited rainfall, and irrigation with marginal water accelerate the accumulation of soluble salts in the root zone [1, 2]. Recent global assessments indicate that more than 1.4 billion hectares of land are affected by salinity and sodicity, with irrigated croplands disproportionately impacted due to poor drainage and prolonged salt build-up [3, 4]. Salinity can reduce average crop yields by 9-20%, depending on stress intensity, soil type, and management practices, making it a critical obstacle to food security under climate change [5, 6]. Excessive salt accumulation imposes multi-faceted injury that includes osmotic stress, ionic toxicity, nutrient imbalance, and oxidative damage, restricting plant growth, development, and final yield [1, 7].

At the cellular level, the toxic accumulation of Na^+^ and Cl^−^ disturbs ion homeostasis, displaces essential cations such as K^+^ and Ca^2+^, and compromises membrane integrity, enzyme function, and metabolic activity [8, 9]. Concurrently, the rise in root-zone osmotic potential restricts water uptake, induces leaf water deficit, and reduces stomatal conductance and CO_2_ assimilation, thereby lowering net photosynthesis [5, 9]. Salinity also triggers the over-production of reactive oxygen species (ROS), which causes lipid peroxidation, protein denaturation, nucleic acid damage, and chlorophyll degradation when not counteracted by efficient antioxidant defenses [7, 10, 11]. Although plants possess multiple tolerance mechanisms; osmotic adjustment, ion compartmentalization, antioxidant defense, and hormonal signaling. These responses are frequently insufficient under prolonged or severe salt stress [11, 12]. Developing sustainable, eco-friendly strategies to mitigate salinity-induced yield losses has therefore emerged as a priority in modern agronomy and plant biotechnology [13].

Maize (*Zea mays* L.) is one of the most economically important cereal crops worldwide, serving as a major source of human food, livestock feed, biofuel, and industrial raw materials, particularly in developing countries [14, 15]. Despite its broad agro-ecological adaptability, maize is classified as a glycophyte with limited intrinsic capacity for Na^+^ exclusion and osmotic adjustment, which renders it especially vulnerable to salt stress [16, 17]. Salinity thresholds as low as ~20 mM NaCl have been reported to compromise maize germination, seedling establishment, root elongation, and biomass accumulation [18, 19]. At advanced growth stages, salinized maize typically displays shorter primary roots, fewer lateral roots, reduced shoot elongation, smaller leaf area, lower canopy coverage, and decreased biomass, reflecting a shift in carbon and energy allocation from vegetative growth toward metabolic stress responses [5, 16, 20], ultimately translating into substantial yield losses [6, 15].

Recent anatomical and ultrastructural studies show that salinity profoundly remodels the architecture of maize roots and leaves. Root tissues, being the first organs in contact with excess salts in the rhizosphere, undergo reductions in root hair density, constriction of the vascular cylinder, and disorganization of xylem vessels, thereby impairing hydraulic conductivity and long-distance water transport [15, 16]. In leaves, salinity reduces palisade and spongy mesophyll thickness, enlarges intercellular air spaces, and disrupts mesophyll organization, compromising light capture, gas exchange, and photosynthetic productivity [16, 21]. At the subcellular level, salt stress can induce plasmolysis, vacuolar membrane rupture, and ultrastructural damage to mitochondria and chloroplasts, producing energy deficits and redox imbalance [5, 22]. Transmission electron microscopy (TEM) is therefore a powerful tool for resolving stress-induced ultrastructural alterations, evaluating membrane and organelle integrity, and locating engineered nanoparticles within plant tissues [22, 23], while complementary anatomical investigation of primary and lateral roots provides essential insight into structural adaptations linked to salinity tolerance [24].

Among biological mitigation strategies, plant growth-promoting rhizobacteria (PGPR) and endophytic bacteria have emerged as promising eco-friendly tools for improving plant performance under saline conditions [25–27]. Endophytic bacteria colonize internal plant tissues without causing disease and establish beneficial associations through phytohormone production, biological nitrogen fixation, phosphate solubilization, siderophore release, 1-aminocyclopropane−1-carboxylate (ACC) deaminase activity, osmolyte accumulation, and induction of host antioxidant defenses [9, 13, 28]. Strains belonging to *Bacillus*, *Pseudomonas*, and *Azospirillum* alleviate salt stress through synergistic mechanisms, including secretion of indole−3-acetic acid (IAA) that enhances root system development and lateral root density, ACC deaminase activity that buffers ethylene-mediated growth suppression, and accumulation of compatible solutes such as proline and glycine-betaine [9, 12, 28]. Inoculation of maize with *Bacillus* sp. PM31 significantly improved agro-morphological traits, chlorophyll content, osmolyte levels, and the activities of antioxidant enzymes such as superoxide dismutase (SOD) and ascorbate peroxidase (APX), while reducing electrolyte leakage and malondialdehyde accumulation under salinity [29]. Similar benefits have been documented in rice, where halotolerant endophytic bacteria modulated ion content, endogenous phytohormones, and the expression of stress-related genes (*OsNHX1*, *OsAPX1*, *OsCATA*) to enhance salinity tolerance [30], and in peanut (*Arachis hypogaea* L.), where endophyte inoculation improved seed germination, root surface area, biomass, and antioxidant enzyme activities under high NaCl concentrations [31]. PGPR-mediated solubilization of phosphorus, potassium, iron, and zinc additionally alleviates the nutritional imbalances caused by salinity [28].

In parallel, nanotechnology has attracted increasing interest in agriculture due to its capacity to improve nutrient delivery, stress tolerance, and crop productivity [23, 32]. Iron is a vital micronutrient that contributes to chlorophyll biosynthesis, photosynthetic electron transport, and numerous enzymatic and metabolic functions; its deficiency markedly limits plant productivity, especially under abiotic stress [29]. Compared with conventional iron fertilizers, iron oxide nanoparticles (FeNPs/Fe_3_O_4_) exhibit superior efficacy owing to their nanoscale dimensions, high specific surface area, enhanced solubility, and improved bioavailability, which together facilitate Fe uptake and translocation in plant tissues [12, 32]. FeNPs can also act as redox catalysts that modulate the activity of enzymatic antioxidants; catalase (CAT), SOD, peroxidase (POD), and ascorbate peroxidase and assist in scavenging excess ROS, thereby reducing oxidative damage to membranes and organelles [11, 16, 33]. In maize, foliar and root application of iron oxide nanoparticles enhanced chlorophyll content, photosynthetic performance, antioxidant enzyme activity, biomass accumulation, and overall tolerance to salinity stress [34], and in wheat, Fe_3_O_4_ nanoparticles improved growth, photosynthesis, and antioxidant activity and modified the distribution of mineral elements under salt stress [35]. Comparable benefits have been reported for FeNPs in spinach under drought and in soybean under cadmium stress, where they reduced ROS accumulation and preserved cellular integrity.

The combined application of beneficial bacteria and engineered nanoparticles represents an emerging frontier in sustainable abiotic-stress management. Co-application of plant growth-promoting bacteria with silica nanoparticles improved the resilience of sugar beet to irrigation with saline water in salt-affected soils [36], while combined treatment with *Providencia vermicola* and iron oxide nanoparticles ameliorated arsenic toxicity in Ajwain (*Trachyspermum ammi* L.) by improving photosynthetic pigments, gas exchange, antioxidant defence, and ultrastructural integrity [37]. The interaction between bacterial cell surfaces and iron oxide nanoparticles is governed by physicochemical processes including electrostatic attraction, hydrogen bonding, van der Waals interactions, and coordination between nanoparticle surfaces and functional groups present in the bacterial cell envelope, such as carboxyl, hydroxyl, phosphate, and amino groups [38]. These interactions facilitate the adsorption and retention of nanoparticles on bacterial surfaces, allowing the bacterial cells to function as efficient carriers for nanoparticle delivery. The stability of the nanoparticle-bacteria association depends on nanoparticle surface properties, bacterial extracellular polymeric substances (EPS), and environmental conditions such as pH and ionic strength [39]. This nano-bio interface may improve the localized availability of iron around colonized root tissues while preserving bacterial colonization efficiency under saline conditions.

Despite extensive independent research on endophytic bacteria and iron oxide nanoparticles (FeNPs) in mitigating abiotic stress, limited information is available regarding their combined application and the resulting nano-bio interactions in maize under salinity stress. In particular, the influence of FeNP-loaded plant growth-promoting endophytic bacteria (PGPEB) on root anatomical organization, ultrastructural integrity, and bacterial localization within plant tissues has not been systematically investigated. A comprehensive set of morphological, physiological, biochemical, and structural parameters was assessed, including germination percentage, growth performance, antioxidant defense responses, root anatomical modifications, and ultrastructural alterations using transmission electron microscopy (TEM). Furthermore, TEM analysis was employed to investigate bacterial colonization patterns and spatial distribution within root tissues under saline conditions. The novelty of this work lies in integrating physiological, anatomical, ultrastructural, and microbiological observations to evaluate the combined application of FeNP-loaded PGPEB under salinity stress. In addition, TEM-based visualization of bacterial localization and nanoparticle distribution within maize root tissues provides further insight into nano-bio interactions under saline conditions.

Collectively, this study contributes to a better understanding of plant-microbe-nanoparticle interactions and highlights the potential of nano-bio formulations as environmentally sustainable tools for improving crop performance under saline conditions.

## Materials and methods

### Plant material and growth conditions

Maize (*Zea mays* L.) grains were obtained from the Agricultural Research Center (ARC), Giza, Egypt; the specific. Grains were visually inspected, and only uniform, undamaged, and disease-free kernels of similar size were retained for the experiments. Plants were maintained throughout the study in a controlled-environment growth chamber operating at a day/night temperature of 25 ± 2 °C / 18 ± 2 °C, a 14 h/10 h light/dark photoperiod, 65 ± 5% relative humidity, and a photosynthetic photon flux density of approximately 350 µmol m^−2^ s^−1^ at canopy level [40].

### Salinity conditioning for endophyte isolation

To recover endophytes pre-adapted to saline conditions, donor maize plants were grown under graded NaCl regimes before root sampling. Surface-sterilized grains were germinated under controlled conditions, and seedlings were irrigated with NaCl solutions at concentrations of 0, 50, 100, 150, or 200 mM throughout the experimental period [31]. Three replicate plants were maintained per NaCl level. At the end of the conditioning phase, root tissues were aseptically harvested and used to isolate salt-tolerant endophytic bacteria.

### Surface sterilization of plant tissues

Surface sterilization was performed to eliminate epiphytic microflora and ensure that only true endophytes were recovered from internal root tissues [41]. Roots were excised, washed thoroughly under running tap water for approximately 10 min, and cut into ~1 cm segments under aseptic conditions in a laminar-flow cabinet. Segments were sequentially immersed in 70% (v/v) ethanol for 1 min, in 2% (w/v) sodium hypochlorite (NaOCl) for 2-3 min, and finally in 70% (v/v) ethanol for ~30 s. The absence of microbial growth after 48 h at 28 °C on plates inoculated with the final rinse water was taken to confirm successful surface sterilization [42].

### Isolation, purification, and screening of salt-tolerant endophytic bacteria

Sterilized root segments (~ 1 cm) were aseptically transferred, without maceration, onto nutrient agar plates and incubated at 28-30 °C for 48 h [43]. Three replicate plates were prepared for each NaCl-conditioning level (50, 100, 150, and 200 mM). Bacterial colonies emerging from the internal tissues of the segments were selected based on distinct morphology (color, form, margin, elevation, opacity) and purified by repeated streaking on fresh nutrient agar until single-colony isolates of uniform appearance were obtained [43]. Pure cultures were maintained on nutrient-agar slants at 4 °C for routine use and preserved in 20% (v/v) glycerol at −80 °C for long-term storage [44]. To select the salt-tolerant isolate for subsequent experiments, bacterial growth and colony abundance were evaluated across the different NaCl-conditioning levels. The highest abundance of bacterial colonies was observed at higher NaCl levels, and a predominant isolate recovered under this condition was selected for subsequent characterization and evaluation of its endophytic colonization capacity in maize seedlings.

### Molecular identification of the selected isolate

The selected isolate was identified by 16S rRNA gene sequencing [45]. Genomic DNA was extracted from an overnight broth culture by a standard CTAB-based protocol. The 16S rRNA gene was amplified by polymerase chain reaction (PCR) using the universal primers 27F (5′-AGAGTTTGATCCTGGCTCAG-3′) and 1492R (5′-GGTTACCTTGTTACGACTT-3′). PCR cycling consisted of an initial denaturation at 95 °C for 5 min, followed by 30 cycles of denaturation at 94 °C for 1 min, annealing at 60 °C for 1 min, and extension at 72 °C for 1 min 30 s, with a final extension at 72 °C for 10 min. Amplicons were purified and Sanger-sequenced commercially. The resulting sequence was submitted to GenBank database to get accession number.

### Preparation of bacterial inoculum

The selected PGPEB isolate was activated by streaking from glycerol stock onto nutrient agar and incubating at 28-30 °C for 24 h. A single colony was transferred to nutrient broth and grown at 28-30 °C with shaking at 150 rpm until the culture reached the late-logarithmic phase (OD₆₀₀ ≈ 0.8-1.0) [46]. Cells were harvested by centrifugation at 6000 × g for 10 min, the supernatant was discarded, and the pellet was washed twice by resuspension in sterilized 0.03 M magnesium sulfate (MgSO₄) and re-centrifugation under the same conditions. The washed pellet was finally resuspended in sterile 0.03 M MgSO₄ and the cell density adjusted to ~1 × 10⁸ CFU mL^−1^, corresponding to a 0.5 McFarland standard, for use as the bacterial inoculum [46].

#### The growth dynamics of the selected PGPEB

Isolates were evaluated in nutrient broth at 28 °C with shaking at 150 rpm. Overnight cultures were inoculated into fresh nutrient broth (1% v/v), and bacterial growth was monitored by measuring optical density at 600 nm (OD_600_) every 2 h for 24 h using a UV–visible spectrophotometer. Three independent cultures were analyzed, and the average OD600 values were used to construct the bacterial growth curve. Cells harvested at the late exponential phase (OD600 ≈ 0.8-1.0) were used for all subsequent experiments.

### Iron oxide nanoparticles and loading on bacterial cells

#### Preparation of FeNP-loaded bacterial cells

Iron oxide (Fe₂O₃) nanoparticle powder obtained from a commercial source was used in this study. The powder was suspended in sterilized deionized water and dispersed by ultrasonication (40 kHz, 30 min) immediately before use to minimize aggregation. The suspension was then diluted to a working concentration of 5 ppm. This concentration was selected based on previously reported physiological effectiveness of Fe₂O₃ nanoparticles in maize seedlings exposed to abiotic stress [47]. In the present study, the nanoparticles were applied via grain soaking to expose the developing seedling internally and assess their effects under systemic uptake conditions.

#### Physicochemical characterization of FeNPs and TEM visualization of FeNP-loaded PGPEB

The physicochemical properties of the commercially obtained FeNPs were evaluated prior to their application. Particle morphology and primary particle size were examined by transmission electron microscopy (TEM) [48]. One drop of the nanoparticle suspension was deposited onto a carbon-coated copper grid, air-dried at room temperature, and examined using a transmission electron microscope operated at an accelerating voltage of 80 kV. The diameters of at least 100 randomly selected nanoparticles were measured using ImageJ software to determine the average particle size and size distribution [49]. The crystalline structure of the FeNPs was analyzed by X-ray diffraction (XRD) using a diffractometer equipped with Cu-Kα radiation (λ = 1.5406 Å). Diffraction patterns were recorded over a 2θ range of 20–80°, and the diffraction peaks were compared with standard reference data to verify the crystalline phase of the iron oxide nanoparticles [50]. The hydrodynamic particle size, polydispersity index (PDI), and zeta potential of the FeNP suspension were determined using a dynamic light scattering (DLS) particle size analyser equipped with electrophoretic light scattering [51]. Prior to analysis, the nanoparticle suspension was ultrasonicated for 10 min to minimize aggregation. The average hydrodynamic diameter, PDI, and zeta potential values were calculated from three independent measurements. The surface chemical functional groups of the FeNPs were characterized by Fourier Transform Infrared Spectroscopy (FTIR) over the spectral range of 4000-400 cm^−1^. The obtained spectra were analysed to identify the characteristic absorption bands corresponding to hydroxyl groups, adsorbed water molecules, and Fe-O bonds [52], thereby confirming the chemical composition of the nanoparticles.

For bacterial observations, unloaded PGPEB cells and FeNP-loaded PGPEB preparations were negatively stained with 2% (w/v) uranyl acetate before TEM examination to visualize the adsorption of FeNPs onto the bacterial cell surface.

#### Adsorption of FeNPs onto PGPEB cells

Equal volumes of the bacterial suspension (1 × 10⁸ CFU mL^−1^) and the 5 ppm FeNPs suspension was mixed and incubated at 28 °C under gentle shaking (100 rpm) for 2 h to allow bacterial adsorption onto the nanoparticles [53]. Successful adsorption of FeNPs onto bacterial cell surfaces was qualitatively confirmed by TEM imaging.

### Seed germination and seedling growth assay

Seed germination and early seedling growth were evaluated under salinity stress to assess the protective effects of PGPEB and FeNPs. The pot experiment was conducted using agricultural clay soil collected from Fayoum, Egypt. Prior to sowing, the soil was air-dried, sieved (2 mm), and analyzed for its physicochemical properties. The soil had a clay texture with a pH of 7.9, electrical conductivity (EC) of 1.46 dS m^−1^, organic matter content of 2.1%, total nitrogen of 0.13%, available phosphorus of 15.2 mg kg^−1^, and available potassium of 268 mg kg^−1^. Each plastic pot (25 cm diameter) contained 2.5 kg of soil and ten maize grains. Three independent pots were used as biological replicates for each treatment. Surface-sterilized maize grains were pre-soaked for 2 h in the respective treatment suspensions and subsequently incubated under controlled conditions. Salinity stress was imposed using NaCl solutions (0-200 mM), maintained throughout the experimental period. Germination was monitored daily for 7 days, and grains were considered germinated upon radicle emergence exceeding 2 mm. The final germination percentage was calculated as $\frac{\mathrm{N}\mathrm{u}\mathrm{m}\mathrm{b}\mathrm{e}\mathrm{r} \mathrm{o}\mathrm{f} \mathrm{g}\mathrm{e}\mathrm{r}\mathrm{m}\mathrm{i}\mathrm{n}\mathrm{a}\mathrm{t}\mathrm{e}\mathrm{d} \mathrm{g}\mathrm{r}\mathrm{a}\mathrm{i}\mathrm{n}\mathrm{s} }{\mathrm{T}\mathrm{o}\mathrm{t}\mathrm{a}\mathrm{l} \mathrm{n}\mathrm{u}\mathrm{m}\mathrm{b}\mathrm{e}\mathrm{r} \mathrm{o}\mathrm{f} \mathrm{s}\mathrm{o}\mathrm{w}\mathrm{n} \mathrm{g}\mathrm{r}\mathrm{a}\mathrm{i}\mathrm{n}\mathrm{s}}\times 100$. After 14 days of growth under the imposed treatments, seedlings were harvested, and morphological traits were recorded. Shoot length was measured from the base of the coleoptile to the tip of the longest leaf, and root length from the base of the coleoptile to the tip of the longest root [30]. At least ten seedlings were measured per replicate, and the replicate mean was used in subsequent analyses.

### Pot experiment

A complementary pot experiment was conducted under greenhouse conditions to validate seedling-level responses in a soil-based system. Maize grains were surface-sterilized, rinsed thoroughly, and randomly assigned to one of five treatments: (i) Control; 0 mM (negative control) and 200 mM NaCl (positive salt-stress control); (ii) PGPEB; grains soaked in the bacterial suspension (~ 1 × 10⁸ CFU mL^−1^); (iii) FeNPs; grains soaked in the 5 ppm Fe₂O₃ nanoparticle suspension; and (iv) PGPEB + FeNPs; grains soaked in the FeNP-loaded PGPEB composite suspension. In all cases, grains were soaked for 2 h with gentle agitation. Pots were arranged in a completely randomized design with three biological replicates per treatment. Each replicate consisted of one pot containing 10 grains. Plants were harvested after 14 d for assessment of growth and physiological responses [30].

### Physiological and biochemical assays

Fresh leaf and root tissues were sampled from harvested seedlings, snap-frozen in liquid nitrogen, and either processed immediately or stored at −80 °C until analysis. All assays were performed in three independent biological replicates, and absorbance was measured on a UV-visible spectrophotometer.

#### Non-enzymatic stress markers

Relative water content (RWC), malondialdehyde (MDA), and free proline were determined in fresh leaf tissues collected from harvested seedlings. RWC was calculated from fresh, turgid, and dry weights [54]. Lipid peroxidation was estimated as MDA equivalents using the thio barbituric-acid-reactive-substances (TBARS) assay according to Heath and Packer [55] and Liang et al. [31]. Free proline content was determined by the acid-ninhydrin method described by Bates et al. [56]. Absorbance measurements were recorded using a UV-visible spectrophotometer, and all assays were performed in three independent biological replicates.

#### Antioxidant enzyme activities

Antioxidant enzyme activities were determined following the salt-stress protocol described by Alharbi et al. [36]. Fresh tissue (0.5 g) was homogenized in cold potassium phosphate buffer and centrifuged at 15,000 × g for 20 min at 4 °C. The resulting supernatant was used for spectrophotometric determination of total soluble protein, superoxide dismutase (SOD), catalase (CAT), and peroxidase (POD) activities. SOD activity was assayed by inhibition of nitro blue tetrazolium photoreduction at 560 nm [57], CAT activity by monitoring H₂O₂ decomposition at 240 nm [58], and POD activity by guaiacol oxidation at 470 nm. Enzyme activities were expressed as U mg^−1^ protein.

#### Phytohormones (IAA and ABA)

Endogenous indole-3-acetic acid (IAA) and abscisic acid (ABA) levels were determined in leaf tissues by HPLC following Nakurte et al. [22]. Hormones were extracted in cold 80% methanol containing butylated hydroxytoluene (BHT), filtered through a 0.45 µm membrane, and separated on a C18 reverse-phase column with UV detection. Hormone concentrations were quantified from external standard curves of authentic IAA and ABA and expressed as ng g^−1^ FW [59].

### Root anatomical analysis

Root anatomical investigations were carried out on primary and lateral roots of harvested plants. Root segments approximately 1-2 cm in length were excised from each treatment, fixed in formalin-acetic acid-alcohol (FAA) solution for 24 h to arrest tissue autolysis, and subsequently preserved in 70% (v/v) aqueous ethanol (ADWIC, Cairo, Egypt) until processing [60]. Transverse cross sections approximately 10-15 µm in thickness were prepared on a REICHERT 820 rotary microtome (Tekyard LLC, Bloomington, CA, USA). Sections were double-stained with safranin and light green (ADWIC, Cairo, Egypt), mounted on glass slides using oven-dried Canada balsam, and examined under a light microscope. Photomicrographs were captured with a 5.1 MP industrial digital camera (Canon, Tokyo, Japan).

### Transmission electron microscopy of root ultrastructure

To examine the ultrastructural effects of salinity and the protective effects of PGPEB and FeNP-loaded PGPEB, small root segments (1–3 mm) were excised from the elongation zone of harvested seedlings and processed for TEM. Samples were fixed in 4% (v/v) glutaraldehyde in 0.2 M sodium phosphate buffer (pH 7.2) for 6-8 h at 4 °C, rinsed in the same buffer, and post-fixed in 1% (w/v) osmium tetroxide for 1 h, followed by additional buffer rinses. Samples were dehydrated through a graded ethanol series (50, 60, 70, 80, 90, 95, and 100%), transitioned through acetone, and embedded in Spurr resin. Ultrathin sections (~ 80 nm) were cut on an ultramicrotome, mounted on copper grids, stained with uranyl acetate and lead citrate, and examined under a transmission electron microscope at an accelerating voltage of 80 kV [10]. Cortical parenchyma, phloem-adjacent cells, and intercellular spaces were inspected for plasmolysis, plasma-membrane integrity, and endophytic bacterial colonization.

### Statistical analysis

Statistical analyses were performed using R software [61]. Data were analyzed using two-way ANOVA to evaluate the effects of salinity, treatment, and their interaction. Mean separation was performed using Tukey's HSD test at *p* < 0.05. Pearson correlation analysis was conducted to investigate relationships among the measured variables. Results are expressed as mean ± standard error (SE).

## Results

### Bacterial isolation and identification

Among the recovered isolates, one predominant colony showed the highest tolerance to NaCl was selected for further characterization. The selected isolate was subjected to Gram staining, followed by phenotypic characterization and subsequent identification. This isolate was then used for further evaluation of its endophytic colonization capacity in maize seedlings. The growth of the bacterial isolate on solid media was used to establish its morphology and Gram staining. Our isolate was Gram-positive and bacilli. PCR was used to separate and amplify the PGPEB isolate's genomic DNA. After being amplified, the 16S rRNA gene segments in the sample were sequenced. PGPEB isolate revealed the closest similarity with *Bacillus* at the genus level. The bacterial isolate PGPEB was identified as *Bacillus* sp. strain PGPR_corkoak_AJ52 16S with maximum homology of 99.41%. The 16S rRNA gene sequences of the bacterial strains were deposited in GenBank database under accession number MZ700077.1

### Growth curve analysis of *Bacillus* sp.

**Fig. 1:**
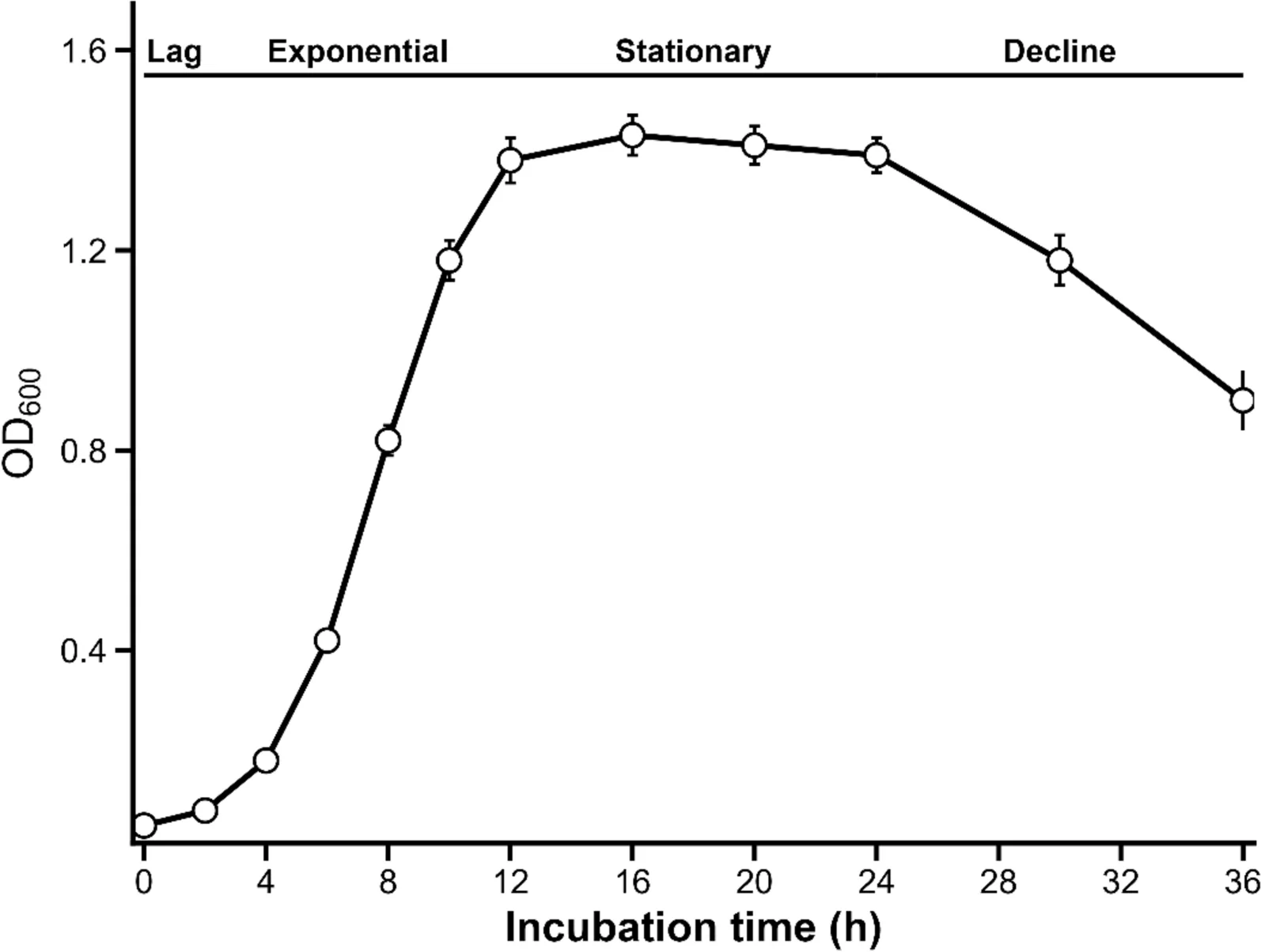
Growth curve of the entophytic *Bacillus* sp. cultured in nutrient broth at 30 °C with shaking at 150 rpm. Optical density (OD₆₀₀) was measured at different incubation times. Values are presented as mean ± SE of three biological replicates

The growth dynamics of the selected endophytic *Bacillus* sp. were evaluated in nutrient broth by monitoring optical density at 600 nm (OD₆₀₀) over a 36 h incubation period (Fig. 1). The isolate exhibited a short lag phase during the first 2 h, followed by a rapid exponential growth phase between 2 and 12 h, reaching a maximum OD₆₀₀ of approximately 1.43 ± 0.04 after 16 h of incubation. Thereafter, the culture maintained a relatively stable stationary phase until 24 h, followed by a gradual decline in cell density after prolonged incubation. Based on these growth characteristics, bacterial cells collected during the late exponential phase were used for FeNPs immobilization and subsequent plant inoculation experiments.

### Physicochemical characterization of FeNPs

#### TEM

**Fig. 2:**
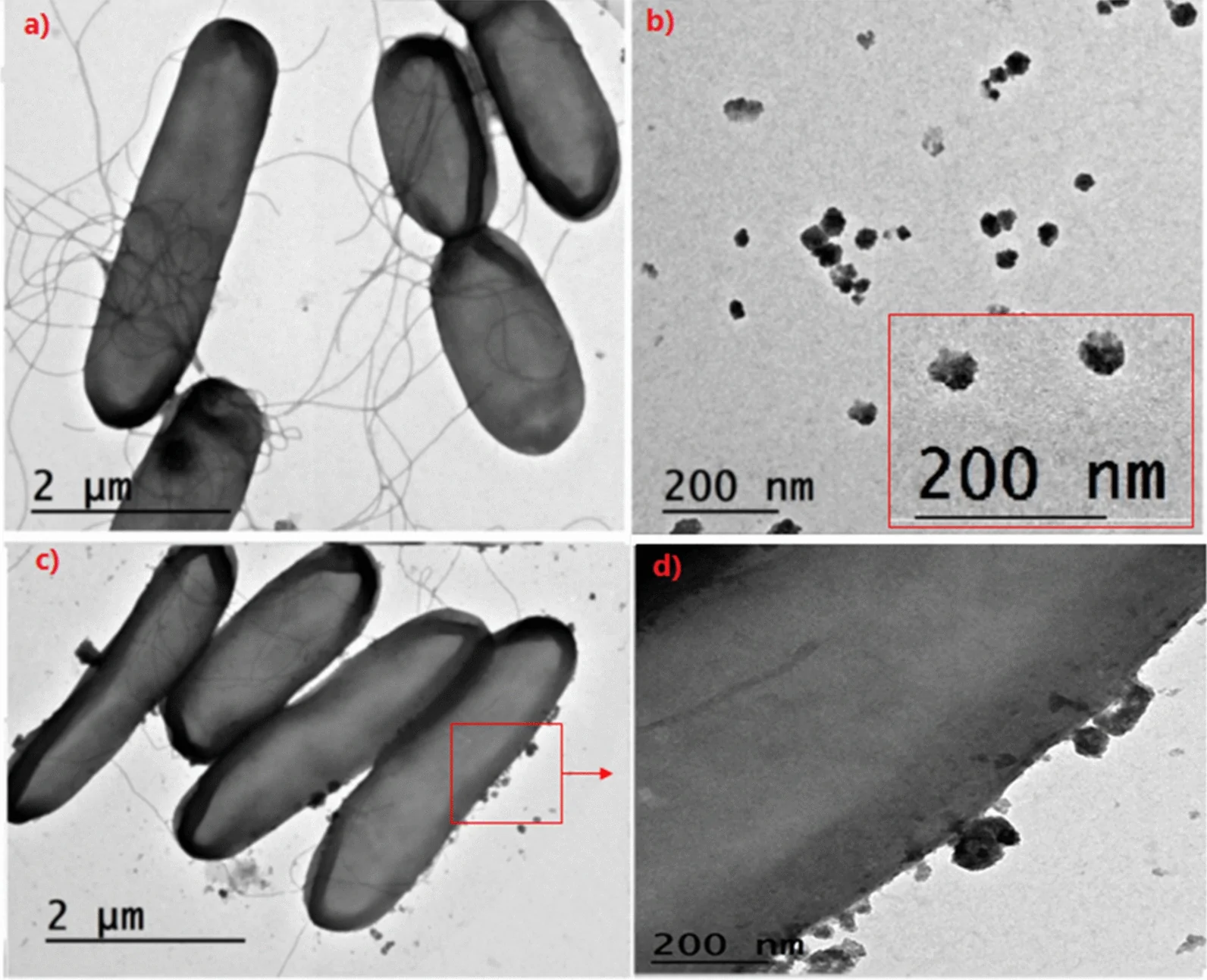
Transmission electron microscopy (TEM) micrographs illustrating the morphology of endophytic bacteria isolated from *Zea mays* roots and their association with iron nanoparticles (FeNPs). **a** Untreated bacterial cells exhibiting rod-shaped morphology with visible flagella, **b** Iron nanoparticles displaying predominantly spherical morphology and nanoscale aggregation patterns; inset shows a magnified nanoparticle cluster, **c** Bacterial cells after interaction/loading with FeNPs, showing nanoparticle attachment on the bacterial surface (highlighted region) and **d** High-magnification TEM image confirming the localization of FeNPs on the bacterial cell envelope

**Fig. 3:**
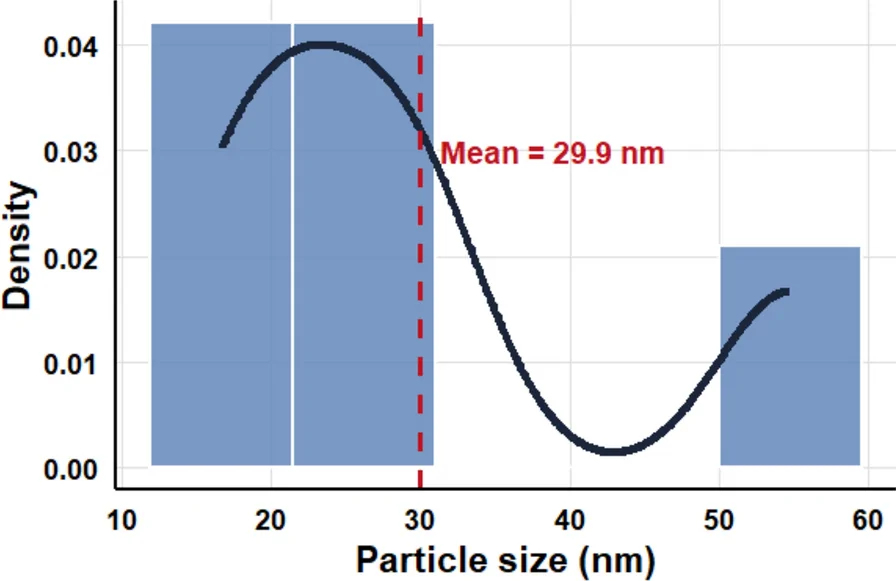
Particle size distribution of FeNPs determined by TEM analysis

The morphology of the endophytic bacterial (PGPEB) isolate obtained from *Zea mays* roots was examined using transmission electron microscopy (TEM). The bacterial cells exhibited a typical rod-shaped morphology with clearly visible flagellar structures, indicating motility and structural integrity (Fig. 2a). Iron nanoparticles (FeNPs) appeared predominantly spherical with nanoscale dimensions and a tendency to form small aggregates (Fig. 2b). Following nanoparticle loading, FeNPs. TEM micrographs confirmed the successful adsorption of FeNPs onto the bacterial cell surface., as evidenced by the presence of electron-dense particles surrounding the bacterial envelope (Fig. 2c, d). The particle size distribution analysis based on TEM micrographs revealed that the synthesized nanoparticles exhibited nanoscale dimensions with an average particle size of approximately 29.9 nm. The distribution curve demonstrated moderate variation in particle diameter, indicating relatively heterogeneous particle populations. Most nanoparticles were distributed within the range of 15-35 nm (Fig. 3). The obtained nanoscale size distribution confirms the successful synthesis of well-dispersed nanoparticles with dimensions.

#### X-ray diffraction (XRD) analysis of FeNPs

**Fig. 4:**
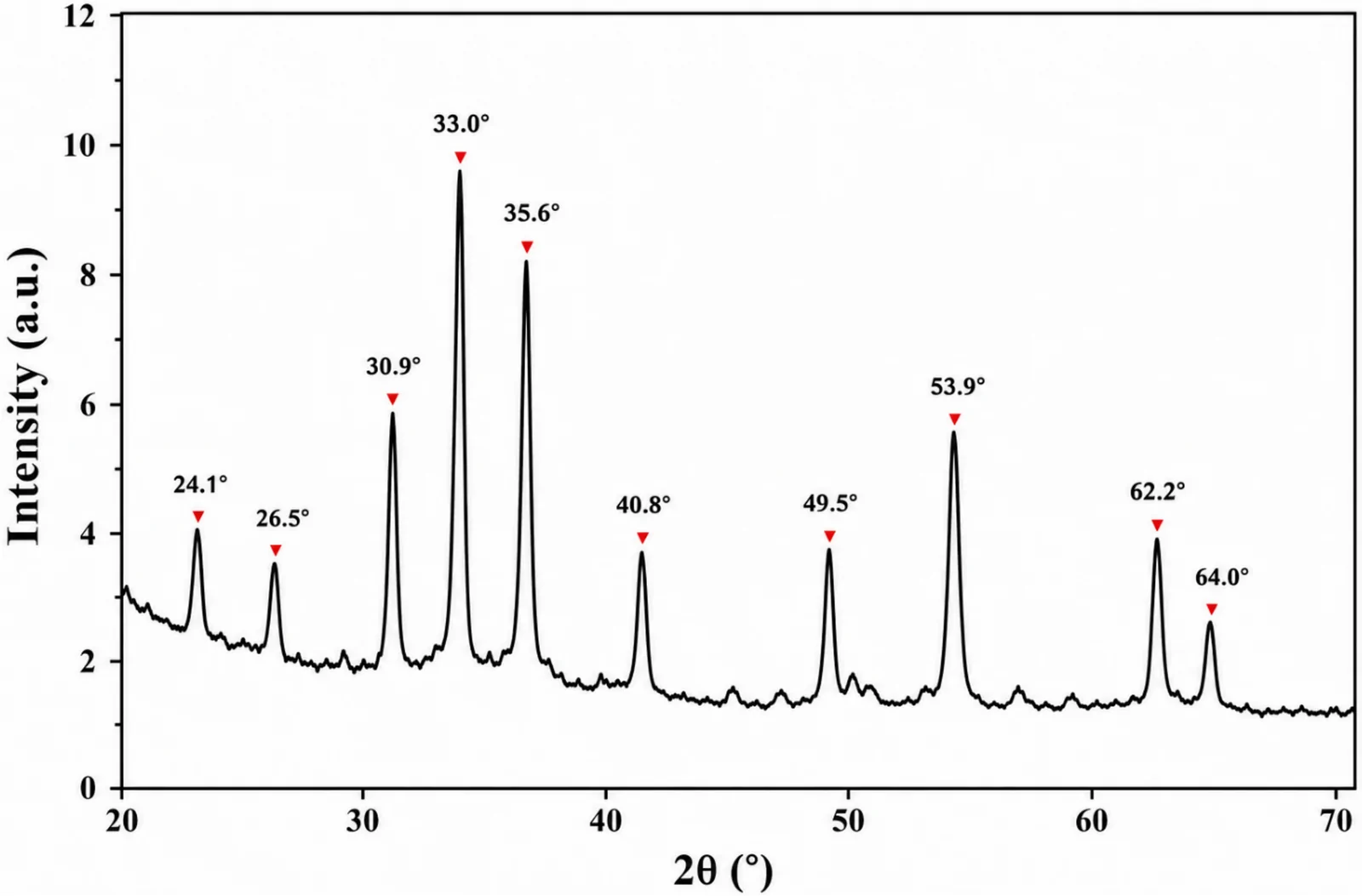
X-ray diffraction (XRD) pattern of the FeNPs shows the characteristic diffraction peaks confirming the crystalline structure and high crystallinity of the nanoparticles

The crystalline structure of the FeNPs was confirmed by X-ray diffraction (XRD) analysis (Fig. 4). The diffraction pattern exhibited distinct and sharp diffraction peaks at 2θ values of approximately 24.1°, 26.5°, 30.9°, 33.0°, 35.6°, 40.8°, 49.4°, 53.9°, 62.3°, and 64.0°, indicating the crystalline nature of the nanoparticles. The most intense diffraction peak was observed at 33.0°, followed by prominent reflections at 35.6° and 53.9°. The presence of these well-defined diffraction peaks confirmed the successful characterization of the FeNPs and demonstrated their high crystallinity with no obvious evidence of amorphous impurities (Fig. 4).

#### Fourier transform infrared spectroscopy (FTIR)

**Fig. 5:**
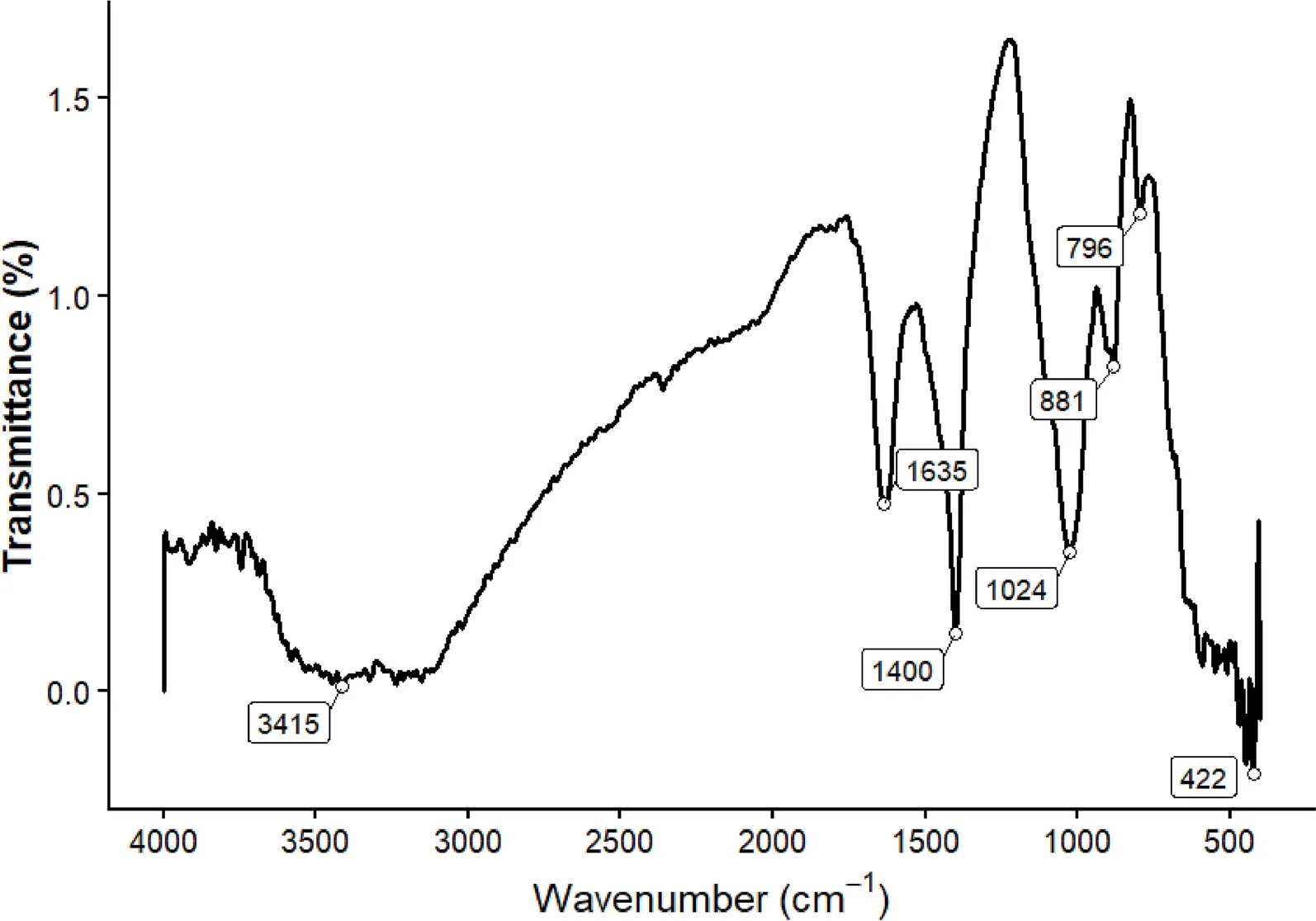
FTIR spectrum of commercially obtained FeNPs shows the characteristic absorption bands corresponding to hydroxyl groups, adsorbed water molecules, and Fe–O vibrations

The FTIR spectrum of the FeNPs (Fig. 5) exhibited several characteristic absorption bands confirming the presence of functional groups associated with iron oxide nanoparticles. A broad absorption band centered at approximately 3416 cm^−1^ corresponded to O–H stretching vibrations, indicating the presence of surface hydroxyl groups and adsorbed moisture. The absorption peak observed at 1636 cm^−1^ was attributed to H–O–H bending vibrations of physically adsorbed water molecules. Additional bands at 1401 and 1025 cm^−1^ were assigned to surface hydroxyl- and C–O-related vibrations. Importantly, characteristic absorption bands detected at approximately 881, 796, and 423 cm^−1^ corresponded to Fe–O stretching vibrations, confirming the formation and crystalline nature of iron oxide nanoparticles.

#### Zeta potential

**Fig. 6:**
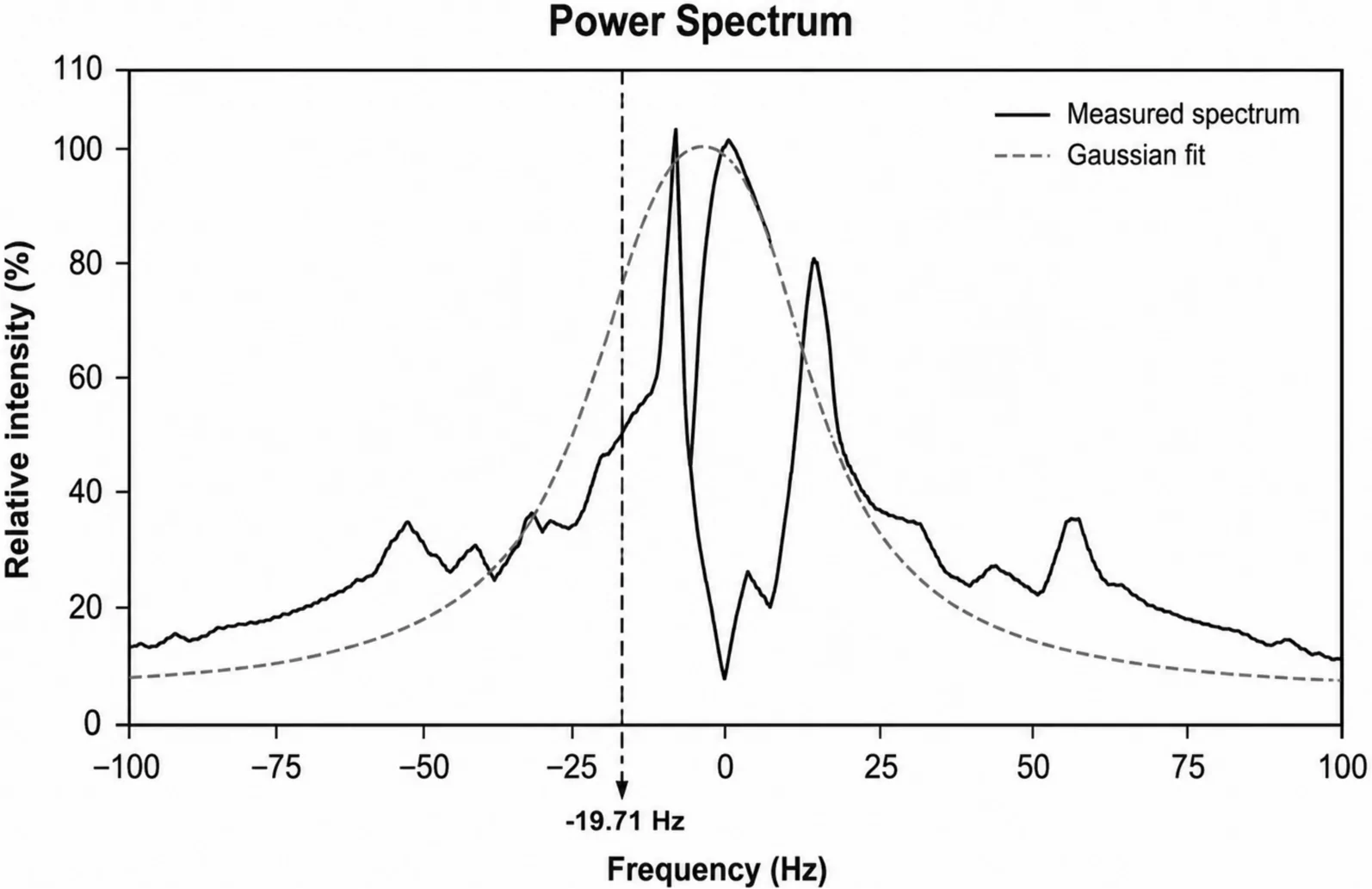
Zeta potential distribution of FeNPs demonstrates a negative surface charge (− 19.71 mV) and moderate colloidal stability

The zeta potential analysis revealed that the commercially obtained FeNPs possessed an average surface charge of − 19.71 mV with an electrophoretic mobility of − 1.47 μm·cm V^−1^ s^−1^ (Fig. 6). The negative zeta potential indicates that the nanoparticle suspension exhibited moderate colloidal stability, suggesting sufficient electrostatic repulsion to reduce excessive particle aggregation under the experimental conditions.

#### Dynamic light scattering (DLS) analysis of FeNPs

**Fig.7:**
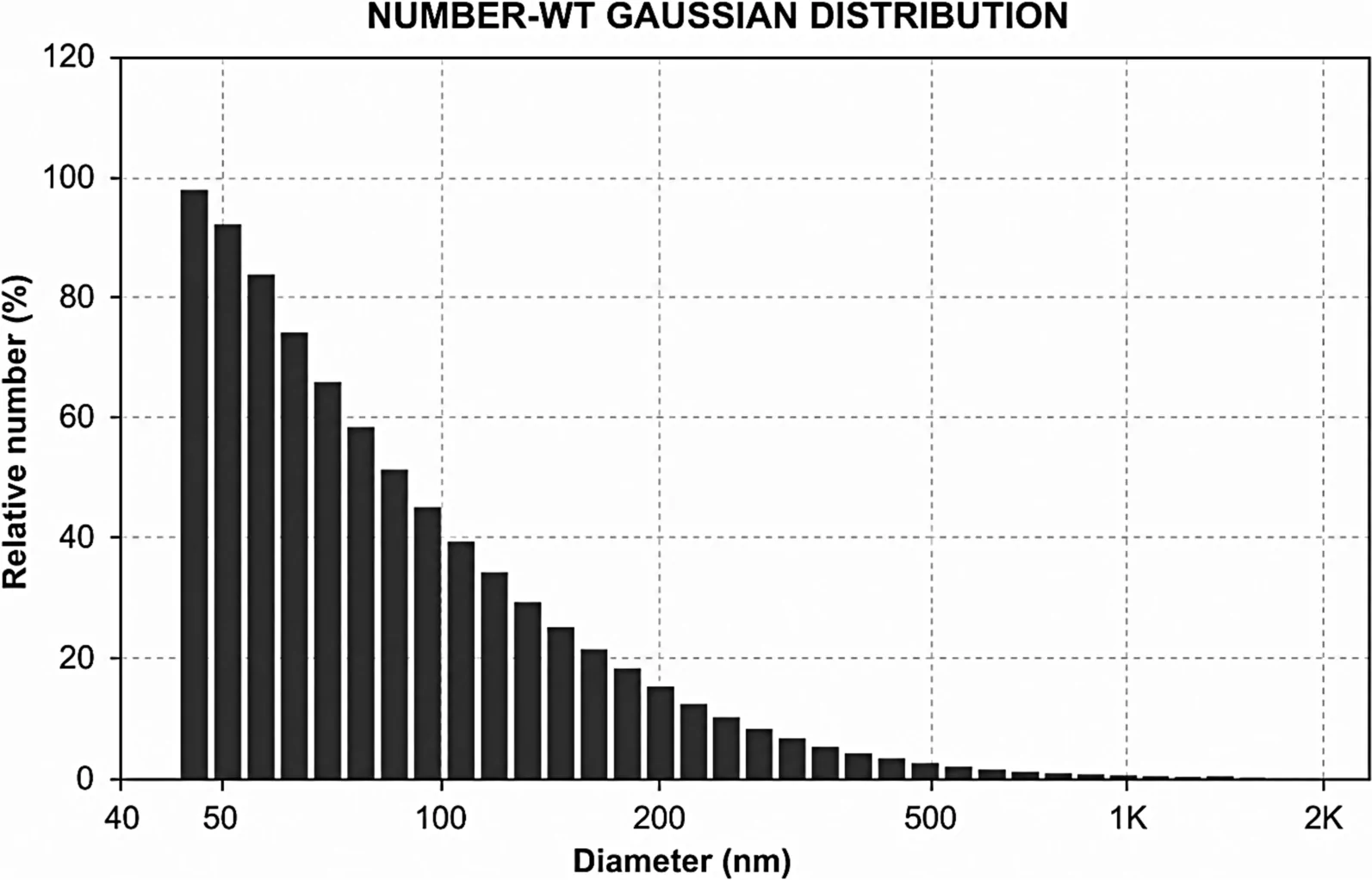
Dynamic light scattering (DLS) analysis shows the hydrodynamic size distribution of FeNPs

Dynamic light scattering (DLS) analysis revealed that the commercially obtained FeNPs exhibited a mean hydrodynamic diameter of 56.8 nm with a polydispersity index (PDI) of 1.100 (Fig. 7). The hydrodynamic particle size determined by DLS was larger than the primary particle size observed by TEM, reflecting the hydrated state of the nanoparticles in suspension and the presence of limited particle aggregation.

### Seedling experiment

#### Effects of PGPEB and FeNPs and salinity on germination rate and plant growth parameters

**Fig. 8:**
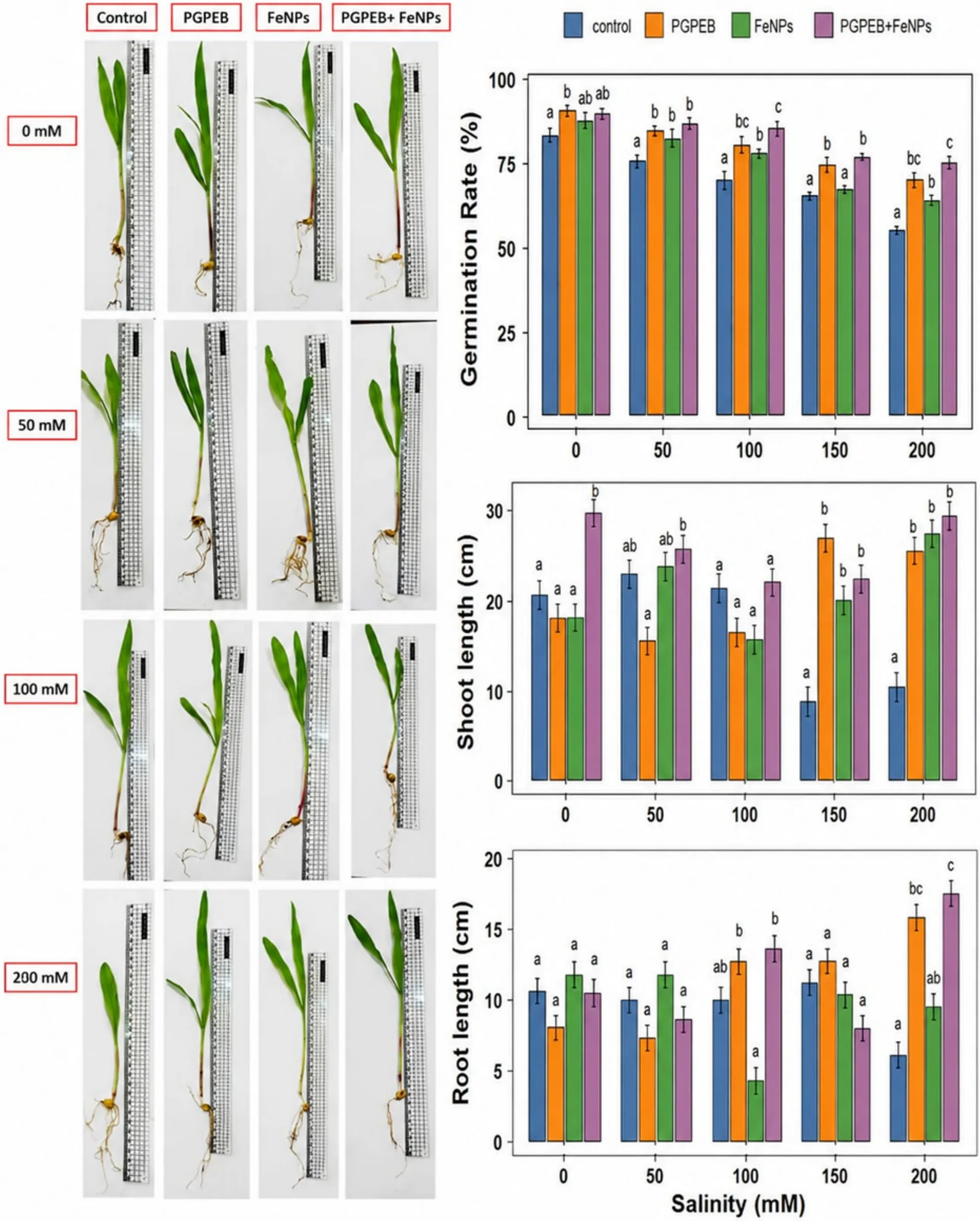
Effect of PGPEB and FeNPs on the growth parameters of *Zea mays* seedlings under salinity stress (0, 50, 100, 150, and 200 mM). **a** Germination rate, **b** shoot length, and **c** root length. Values are presented as means of three replicates ± standard error. Data were analyzed using two-way ANOVA to assess differences among treatments. Different letters indicate statistically significant differences among treatments within each salinity level (*P* ≤ 0.05)

Salinity stress significantly reduced the germination rate of *Zea mays* across all treatments (Fig. 8a), with the lowest values observed in untreated plants at 200 mM NaCl. However, PGPEB, FeNPs, and particularly the FeNP-loaded PGPEB treatment improved germination under saline conditions compared with the control. FeNP-loaded PGPEB treatment consistently showed the highest germination percentages at moderate and high salinity levels (100&200 mM), indicating an enhanced beneficial effect of the combined PGPEB + FeNPs treatment in alleviating salt stress. FeNPs alone also enhanced germination under salinity, whereas PGPEB showed moderate improvement. Shoot length was significantly affected by salinity and treatment application (Fig. 8b). Salinity stress markedly reduced shoot growth in untreated plants, particularly at 150 and 200 mM NaCl. In contrast, PGPEB, FeNPs, and especially the FeNP-loaded PGPEB treatment improved shoot length under saline conditions.

FeNP-loaded PGPEB treatment consistently produced the highest shoot length values across most salinity levels, indicating enhanced tolerance to salt stress. PGPEB alone also showed a strong promotive effect at higher salinity levels, while FeNPs treatment moderately improved shoot growth compared with the untreated control. Root length was significantly influenced by the interaction between salinity and treatment (Fig. 8c). Increasing salinity reduced root growth in untreated plants, particularly at 200 mM NaCl. However, PGPEB, FeNPs, and FeNP-loaded PGPEB improved root length under saline conditions compared with the control. FeNP-loaded PGPEB exhibited the highest root length under severe salinity (200 mM), indicating enhanced root adaptation and stress tolerance. PGPEB alone also markedly promoted root elongation under high salinity, whereas FeNPs treatment showed moderate improvement.

### Effect of PGPEB and FeNPs treatments on enzymatic and nonenzymatic antioxidants

**Fig. 9:**
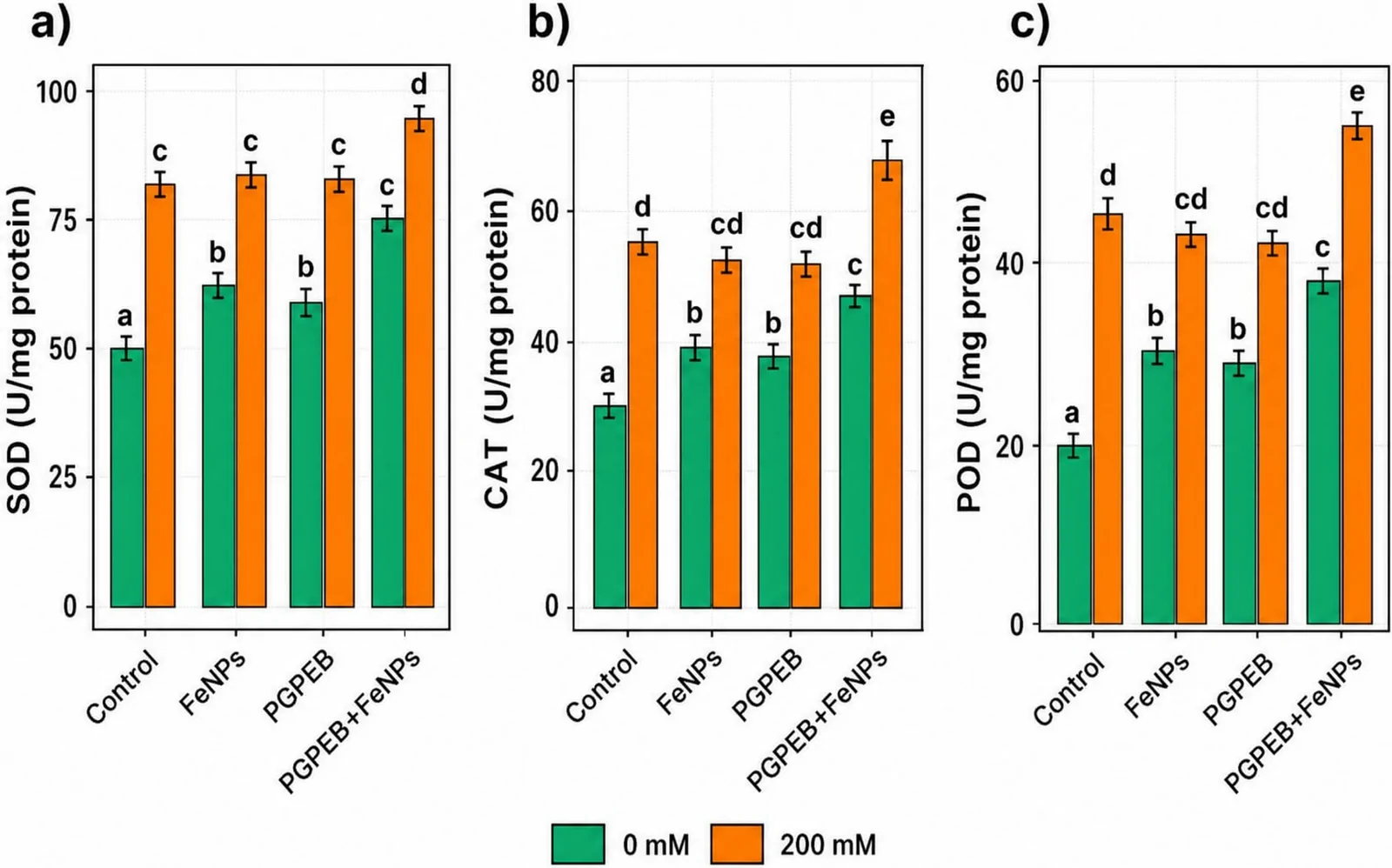
Effects of endophytic plant growth-promoting bacteria (PGPEB) and iron nanoparticles (FeNPs) on antioxidant enzyme activities in Zea mays under salinity stress conditions. **a** Superoxide dismutase (SOD), **b** catalase (CAT), and **c** peroxidase (POD) activities in maize plants grown under 0 and 200 mM NaCl. Different letters above bars indicate significant differences among treatments according to Tukey’s post hoc test at *P* ≤ 0.05

Salinity stress significantly reduced the activities of SOD, CAT, and POD enzymes in *Zea mays* across all treatments (Fig. 9), with the lowest values observed in untreated plants at 200 mM. However, FeNPs, PGPEB, and the FeNP-loaded PGPEB treatment exhibited the highest SOD, CAT, and POD activities under salinity stress compared with the control, indicating combined effect between the bacterial inoculation and iron nanoparticles in improving oxidative stress tolerance. Under non-saline conditions (0 mM NaCl), treated plants also showed moderate increases in antioxidant enzyme activities compared with the untreated control, suggesting that PGPEB and FeNPs stimulated basal antioxidant metabolism even in the absence of stress. However, the magnitude of enhancement was substantially greater under salinity stress, emphasizing the stress-responsive protective role of these treatments. The elevated antioxidant enzyme activities observed in PGPEB and FeNP-treated plants may contribute to more efficient detoxification of reactive oxygen species (ROS), stabilization of cellular membranes, and maintenance of cellular redox balance under saline conditions. The superior performance of the combined treatment suggests that the interaction between endophytic bacteria and FeNPs improved the physiological adaptation of maize plants to high salinity stress.

**Fig. 10:**
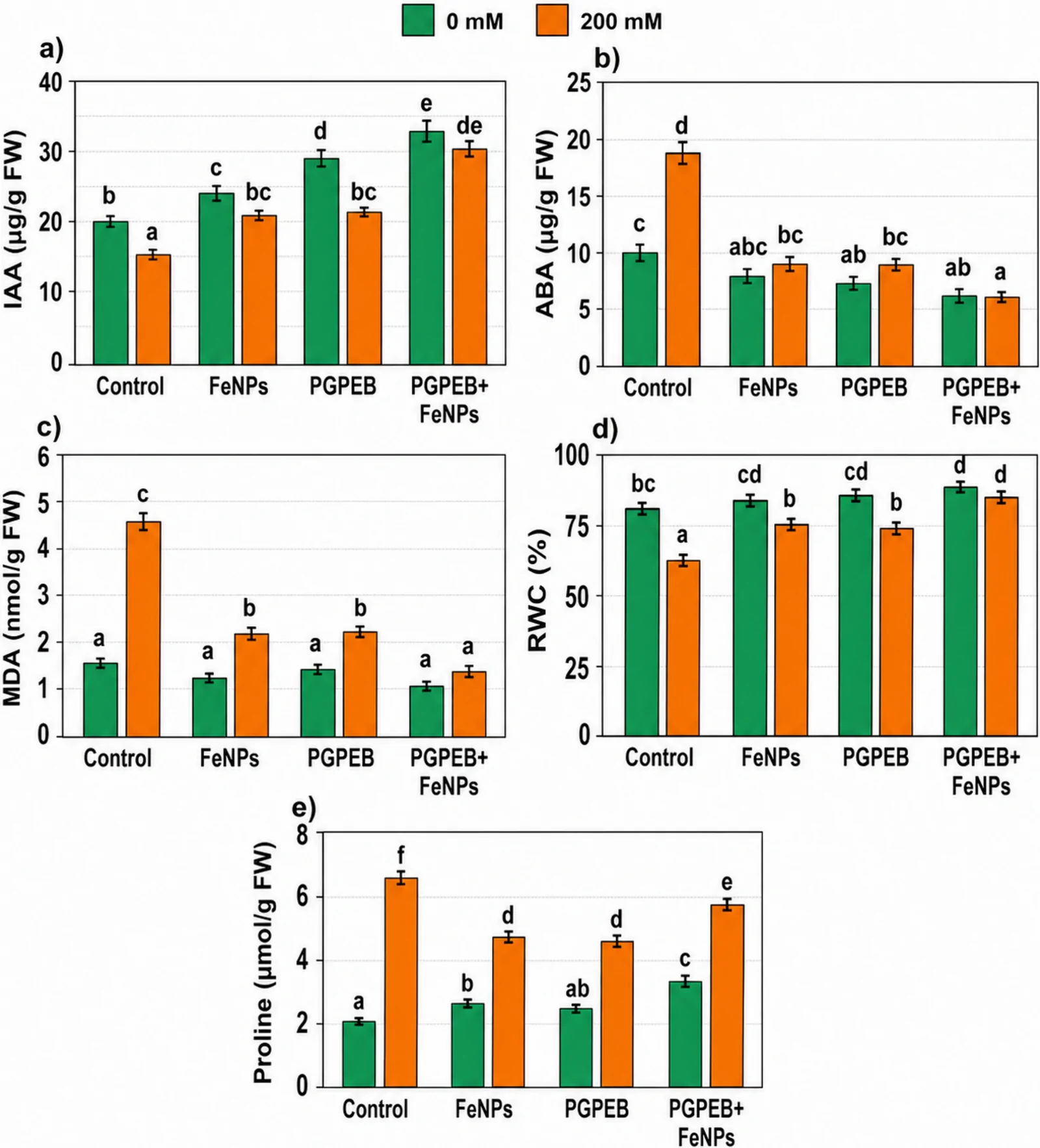
Effects of endophytic plant growth-promoting bacteria (PGPEB) and iron nanoparticles (FeNPs) on physiological and biochemical responses of *Zea mays* under salinity stress conditions. **a** Indole-3-acetic acid (IAA), **b** abscisic acid (ABA), **c** malondialdehyde (MDA), **d** relative water content (RWC), and **e** proline content in maize plants grown under 0 and 200 mM NaCl. Different letters above bars indicate significant differences among treatments according to Tukey’s post hoc test at *P* ≤ 0.05

The physiological and biochemical responses of *Zea mays* plants were significantly affected by salinity stress and the applied treatments (Fig. 10). Salinity stress (200 mM NaCl) markedly altered the levels of indole-3-acetic acid (IAA), abscisic acid (ABA), malondialdehyde (MDA), relative water content (RWC), and proline accumulation, indicating substantial metabolic and osmotic adjustments under stress conditions. IAA content showed a general enhancement in treated plants compared with the untreated control, particularly under the FeNP-loaded PGPEB treatment, which exhibited the highest IAA levels under both normal and saline conditions. This suggests that endophytic bacteria and iron nanoparticles promoted phytohormonal balance and stimulated plant growth even under salt stress. In contrast, ABA levels increased considerably in salt-stressed control plants, reflecting activation of stress signaling pathways. However, plants treated with PGPEB and FeNPs exhibited comparatively lower ABA accumulation under salinity, indicating partial alleviation of stress perception and improved physiological adaptation. Lipid peroxidation, estimated by MDA content, was strongly elevated under salinity stress in untreated plants, confirming severe oxidative membrane damage. Application of PGPEB and FeNPs significantly reduced MDA accumulation compared with salt-stressed controls, demonstrating their protective role in minimizing oxidative injury and preserving membrane integrity. The lowest MDA levels under salinity were observed in the FeNP-loaded PGPEB treatment, indicating superior stress mitigation efficiency. Relative water content (RWC) declined markedly in salt-stressed control plants, reflecting impaired water status and osmotic imbalance. Nevertheless, treated plants maintained significantly higher RWC values under salinity, particularly with the FeNP-loaded PGPEB treatment, suggesting improved water retention capacity and enhanced osmotic adjustment. Proline accumulation increased substantially under salinity stress across all treatments, consistent with its role as an osmoprotectant under adverse environmental conditions. The highest proline content was recorded in salt-stressed plants treated with FeNP-loaded PGPEB, indicating enhanced osmotic protection and stress tolerance mechanisms.Overall, the FeNP-loaded PGPEB was the most effective treatment in improving hormonal balance, reducing oxidative damage, maintaining plant water status, and enhancing osmoprotective responses in maize plants subjected to high salinity stress.

### PCA-based discrimination of maize responses under salinity stress

**Fig. 11:**
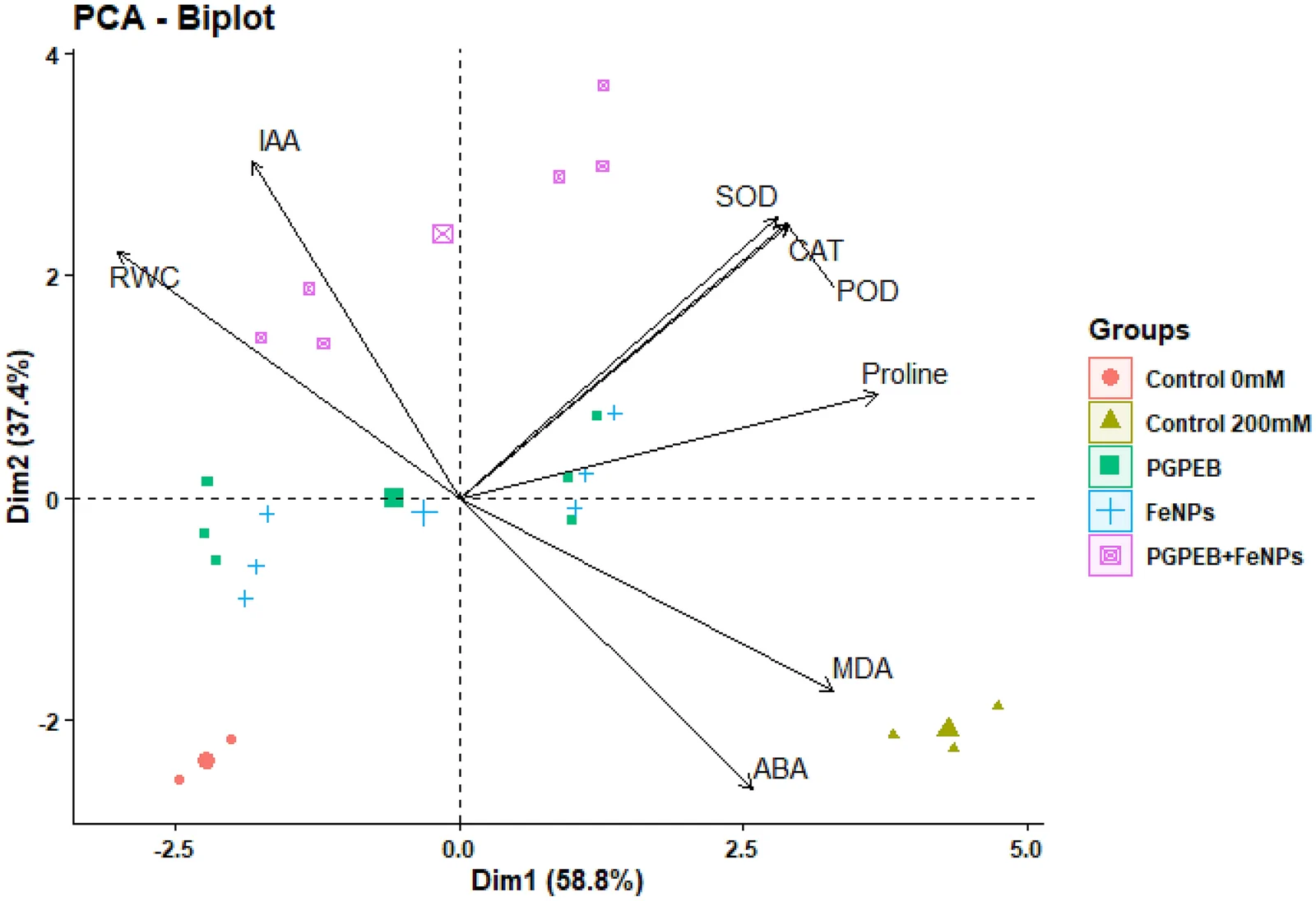
Principal component analysis (PCA) revealed a clear separation among treatments under salinity stress conditions based on the evaluated physiological and biochemical parameters. The first two principal components explained 96.2% of the total variation, with PC1 and PC2 accounting for 58.8% and 37.4% of the variance, respectively, indicating strong discrimination among treatments

Salt-stressed control plants (Control 200 mM) were positively associated with ABA and MDA, reflecting enhanced oxidative stress and stress signaling under salinity conditions (Fig. 11). In contrast, PGPEB and FeNPs-treated plants were more closely associated with antioxidant enzymes (SOD, CAT, and POD), indicating improved antioxidant defense responses. The FeNP-loaded PGPEB treatment showed the closest association with proline accumulation and antioxidant activities, suggesting enhanced osmotic adjustment and stress tolerance under salinity stress. Meanwhile, the non-saline control (Control 0 mM) was positively correlated with RWC and IAA, indicating favorable water status and growth-promoting hormonal balance under normal conditions. Overall, the PCA results demonstrated that FeNP-loaded PGPEB substantially modified the physiological and biochemical responses of maize plants, contributing to improved adaptation to salinity stress.

### Ultrastructural analysis of maize root cells under salinity stress

**Fig. 12:**
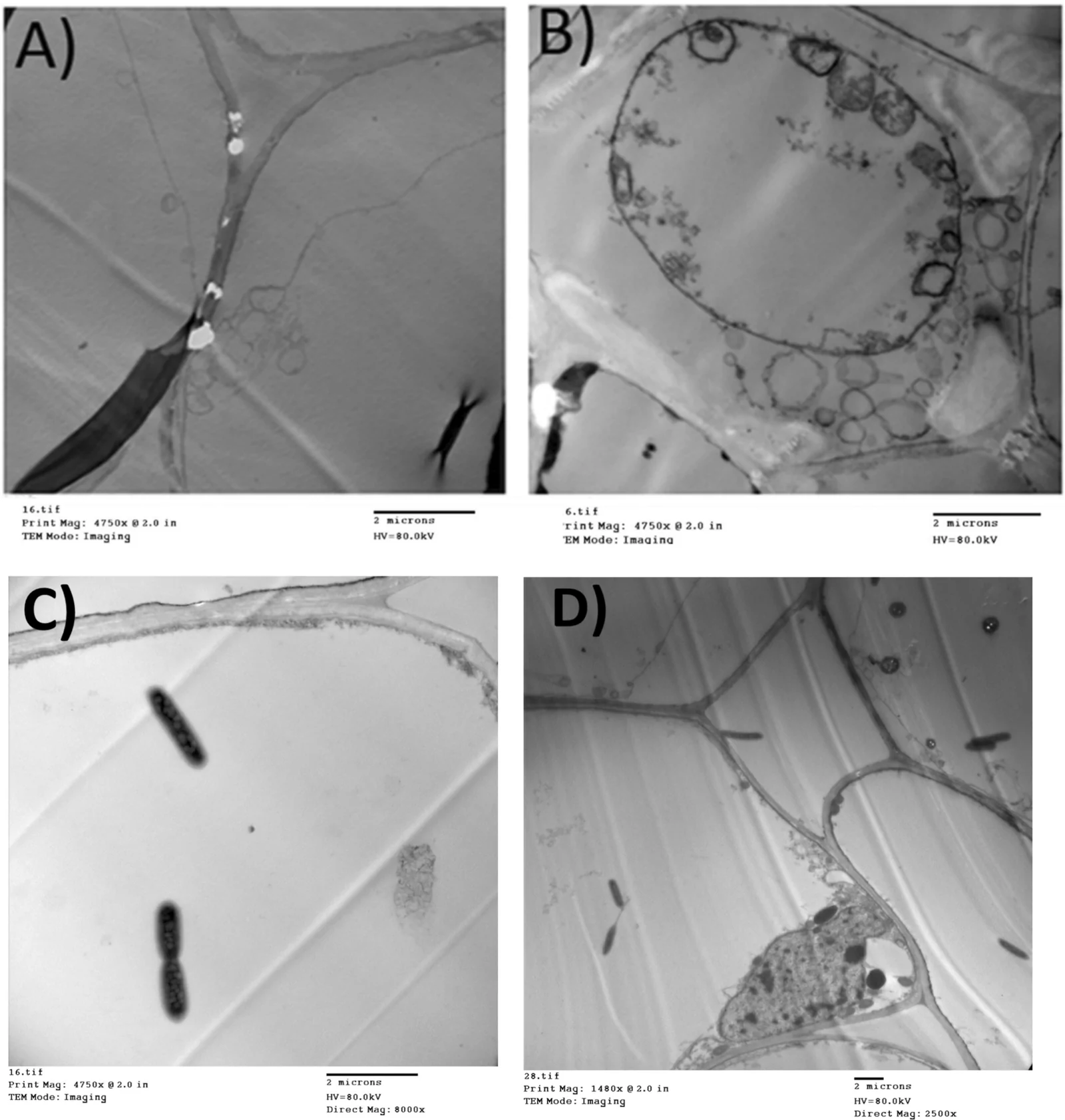

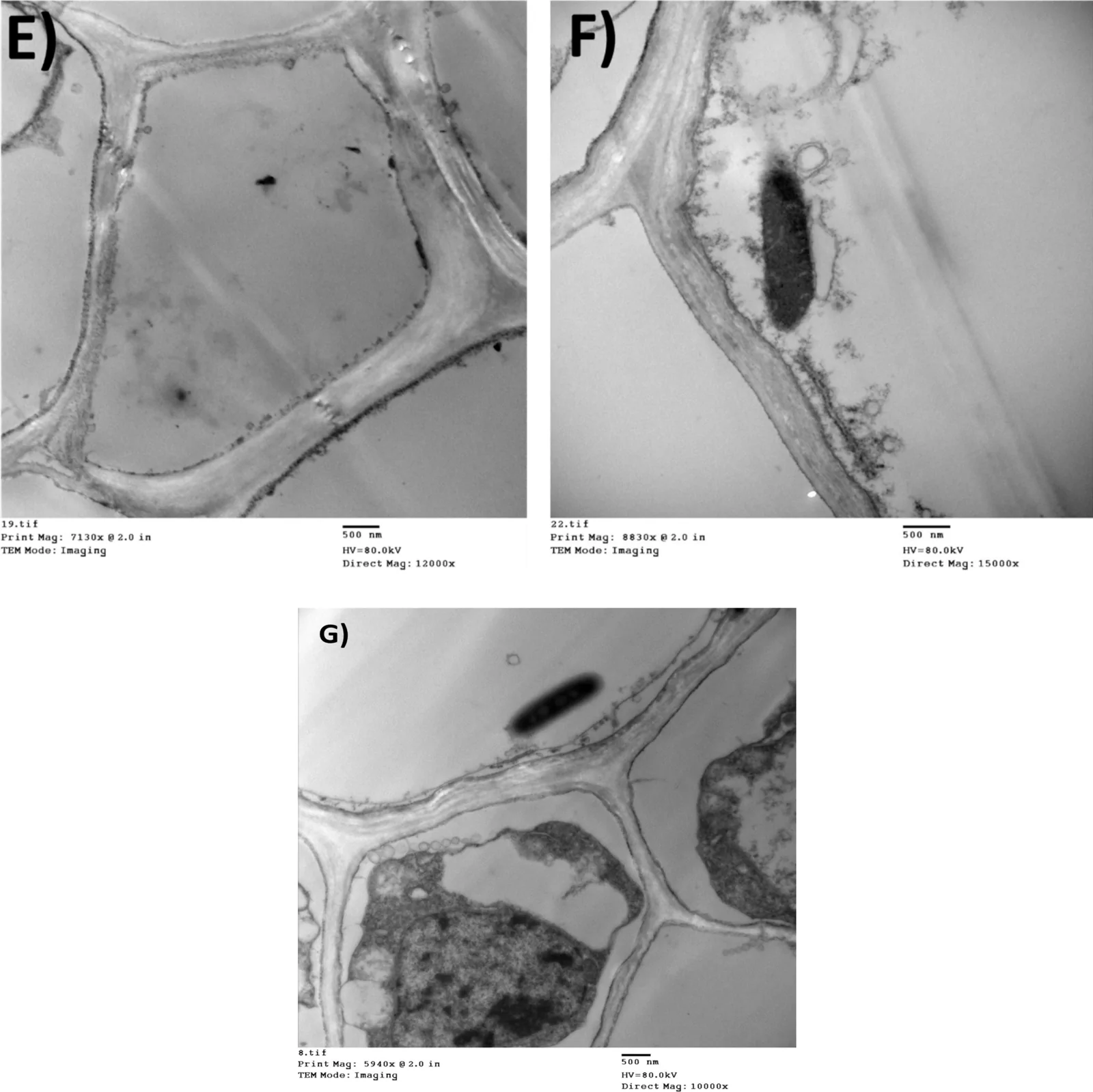
Transmission electron microscopy (TEM) micrographs illustrating ultrastructural alterations and tissue-specific localization of endophytic bacteria in *Zea mays* roots under salinity stress and different treatments. **A** Control root cortical cells show no plasmolysis, with intact cell wall, continuous plasma membrane, and dense, well-organized cytoplasm. **B** Cells exposed to 200 mM NaCl show pronounced plasmolysis, characterized by plasma membrane detachment from the cell wall, protoplast shrinkage, and formation of electron-transparent spaces. **C** PGPEB-treated roots under salinity stress shows partial reduction of plasmolysis, with plasma membrane mostly attached to the cell wall and limited cytoplasmic shrinkage. Bacterial cells are observed within cells, confirming endophytic colonization. **D** Vascular-associated (phloem-adjacent) tissue shows localized bacterial colonization near elongated cells. **E** Roots treated with nanoparticles under salinity stress shows minimal plasmolysis, with preserved plasma membrane-cell wall continuity and maintained cytoplasmic integrity. **F** Roots treated with nanoparticles-loaded bacteria under salinity stress shows no plasmolysis. **G** Cortical parenchymatous tissue shows heterogeneous bacterial colonization. Some cells contain bacterial cells (minimal plasmolysis), while adjacent cells show no detectable bacteria (maximal plasmolysis was observed)

Transmission electron microscopy (TEM) revealed clear ultrastructural alterations in maize root tissues under salinity stress and different treatments, with emphasis on plasmolysis and plasma membrane integrity. The examined sections included cortical and vascular tissues (Fig. 12), particularly cortical parenchyma and phloem-associated cells, which differed in their structural response and bacterial colonization patterns. Control cells showed no plasmolysis, with intact cell walls, continuous plasma membranes, and dense cytoplasm indicating normal cellular organization. Exposure to 200 mM NaCl induced pronounced plasmolysis, characterized by plasma membrane detachment from the cell wall, protoplast shrinkage, and formation of electron-transparent spaces, reflecting severe osmotic stress. In PGPEB-treated roots under salinity stress, plasmolysis was partially reduced, as most cells-maintained plasma membrane attachment or showed only slight detachment. Bacterial cells were observed within intercellular spaces, confirming successful colonization. In roots treated with nanoparticles-loaded bacteria under salinity stress, plasmolysis was minimal or absent in most cells, with preserved plasma membrane-cell wall continuity and maintained cytoplasmic structure. Cortical parenchymatous tissue exhibited heterogeneous bacterial colonization, where some cells contained bacterial aggregates associated with the cell wall, while adjacent cells lacked detectable bacteria. Variable degrees of plasmolysis were also observed among neighboring cells within the same tissue region. Vascular-associated (phloem-adjacent) tissue showed localized bacterial colonization near elongated cells, with some cells colonized and others uncolonized. Cells in this region displayed relatively preserved ultrastructure with maintained wall integrity and moderate cytoplasmic organization under nanoparticles-loaded bacteria treatment.

### Anatomical alterations in primary and lateral roots of *Zea mays* under salinity stress and the protective effects of FeNP-loaded bacteria

**Fig. 13:**
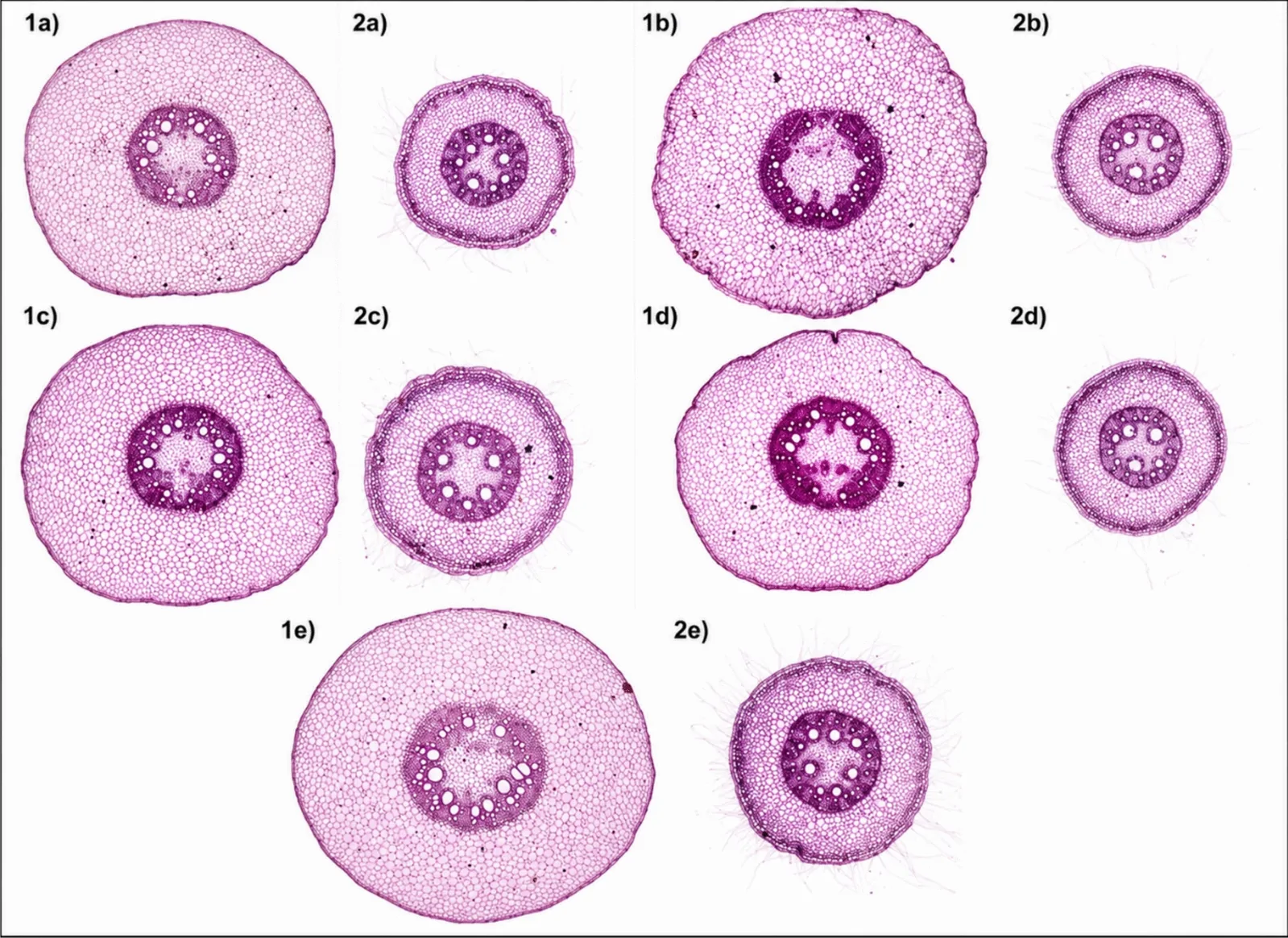
Anatomical transverse sections of primary and lateral roots of *Zea mays* under different treatments. **a** Healthy control root under non-saline conditions (0 mM NaCl), **b** Root exposed to 200 mM salinity stress, **c** Salt-stressed root treated with plant growth-promoting endophytic bacteria (PGPEB), **d** Salt-stressed root treated with iron nanoparticles (FeNPs) and **e** Salt-stressed root treated with FeNPs-loaded bacteria. Structural variations in vascular tissues, parenchyma cells, supporting tissues, and root organization were observed among treatments

Microscopic examination of transverse sections of primary and lateral roots of *Zea mays* revealed marked anatomical variations among the investigated treatments under salinity stress conditions (200 mM NaCl) (Fig. 13). Salinity stress induced considerable structural modifications in root tissues compared with healthy control plants, whereas treatment with plant growth-promoting endophytic bacteria (PGPEB), iron nanoparticles (FeNPs), and FeNPs-loaded bacteria alleviated many of these deleterious effects.

#### Primary root anatomy

Qualitative observations demonstrated that healthy control roots exhibited a rounded section outline with few slight ribs and a well-organized supporting tissue ring. Exposure to salinity stress resulted in slight deformation of the section outline and supporting tissues, indicating stress-induced structural alterations. In contrast, PGPEB and FeNPs-loaded bacteria treatments restored a more regular rounded morphology, suggesting improved structural stability under saline conditions. Quantitative anatomical analysis further confirmed the adverse effect of salinity stress on root tissues (Table 1). Salinity treatment increased root diameter from 0.882 mm in control plants to 1.02 mm, while reducing parenchyma thickness, phloem thickness, and xylem vessel diameter. Salinity stress also decreased the number of xylem vessels and reduced the distance between pith and vascular cylinder, indicating vascular compression and impaired conductive tissue organization. Application of PGPEB under salinity stress enhanced several anatomical parameters compared with salt-stressed plants, including phloem thickness (0.046 mm), xylem vessel number (11 vessels), and parenchyma row number (17 rows), reflecting improved tissue development and vascular differentiation. Similarly, FeNP treatment partially restored vascular organization and increased collenchyma thickness relative to salt-stressed plants. The FeNPs-loaded bacteria treatment exhibited the greatest improvement in root anatomy. This treatment increased parenchyma thickness (0.290 mm), vascular bundle width (0.101 mm), vascular cylinder thickness (0.139 mm), xylem tissue thickness (0.093 mm), and collenchyma thickness (0.022 mm) compared with salinity-stressed roots. Moreover, the number of sclerenchyma vascular cylinder sheath rows increased to three rows under FeNPs-loaded bacterial treatment, indicating enhanced mechanical support and tissue reinforcement.

**Table 1:** Comparative analysis of primary root anatomical traits under salinity stress and biostimulant/nanoparticle treatments

Character	Control (0 mM)	Control (200 mM)	PGPEB	FeNPs	PGPEB + FeNPs	*p*-value
Root diameter (mm)	0.854 ± 0.006^a^	1.011 ± 0.007^b^	0.896 ± 0.011^b^	0.830 ± 0.011^a^	1.017 ± 0.012^c^	< 0.05
Parenchyma thickness (mm)	0.260 ± 0.002^bc^	0.262 ± 0.001^c^	0.227 ± 0.002^b^	0.212 ± 0.002^a^	0.288 ± 0.003^d^	< 0.05
Number of parenchyma rows	14.04 ± 0.16^b^	13.03 ± 0.13^a^	17.29 ± 0.18^d^	14.92 ± 0.22^bc^	13.88 ± 0.34^c^	< 0.05
Distance between pith and vascular cylinder (mm)	0.196 ± 0.003^d^	0.082 ± 0.001^a^	0.167 ± 0.002^c^	0.170 ± 0.002^c^	0.150 ± 0.002^b^	< 0.05
Phloem thickness (mm)	0.032 ± 0.000^a^	0.025 ± 0.000^a^	0.045 ± 0.001^c^	0.041 ± 0.001^c^	0.037 ± 0.000^b^	< 0.05
Xylem vessel diameter (mm)	0.037 ± 0.000^b^	0.030 ± 0.000^a^	0.033 ± 0.000^a^	0.033 ± 0.000^a^	0.040 ± 0.000^b^	< 0.05
Xylem tissue thickness (mm)	0.078 ± 0.002^b^	0.088 ± 0.001^c^	0.079 ± 0.002^b^	0.067 ± 0.001^a^	0.092 ± 0.001^c^	< 0.05
Number of xylem vessels	10.02 ± 0.10^b^	8.59 ± 0.13^a^	11.14 ± 0.10^c^	8.04 ± 0.06^a^	10.86 ± 0.14^c^	< 0.05
Vascular cylinder width (mm)	0.074 ± 0.001^a^	0.074 ± 0.000^b^	0.068 ± 0.001^a^	0.077 ± 0.001^a^	0.101 ± 0.002^c^	< 0.05
Vascular cylinder thickness (mm)	0.107 ± 0.002^b^	0.092 ± 0.001^a^	0.132 ± 0.002^c^	0.105 ± 0.001^a^	0.137 ± 0.002^c^	< 0.05
Number of vascular cylinder rings	1.00 ± 0.00^a^	1.00 ± 0.00^a^	1.00 ± 0.00^a^	1.00 ± 0.00^a^	3.06 ± 0.05^b^	< 0.05
Number of pith parenchyma rows	7.00 ± 0.14^a^	5.00 ± 0.10^b^	7.00 ± 0.14^a^	8.00 ± 0.16^c^	7.00 ± 0.14^a^	< 0.05
Collenchyma thickness (mm)	0.014 ± 0.000^c^	0.010 ± 0.000^a^	0.012 ± 0.000^b^	0.015 ± 0.000^d^	0.022 ± 0.000^e^	< 0.05
Number of collenchyma rows	2.00 ± 0.00^a^	2.00 ± 0.00^a^	2.00 ± 0.00^a^	2.00 ± 0.00^a^	2.00 ± 0.00^a^	> 0.05
Number of sclerenchyma sheath rows around vascular cylinder	1.00 ± 0.01^a^	0.99 ± 0.02^a^	1.99 ± 0.04^b^	1.01 ± 0.01^a^	3.06 ± 0.05^c^	< 0.05

#### Lateral root anatomy

Similar anatomical trends were observed in lateral roots (Table 2). Salinity stress altered tissue organization and affected vascular characteristics compared with the healthy control. Salt-treated roots exhibited increased xylem tissue thickness and vascular cylinder width, accompanied by changes in pith parenchyma arrangement. Treatment with PGPEB enhanced root diameter (0.660 mm), parenchyma thickness (0.159 mm), and vascular tissue organization relative to salt-stressed plants. FeNP application also improved vascular cylinder characteristics and maintained xylem tissue integrity under salinity stress. The FeNPs-loaded bacterial treatment produced the most pronounced anatomical recovery in lateral roots, resulting in the highest root diameter (0.671 mm), increased vascular cylinder width (0.057 mm), improved phloem thickness (0.038 mm), and enhanced sclerenchyma development compared with untreated salt-stressed roots (Fig. 13).

**Table 2:** Comparative analysis of lateral root anatomical traits under salinity stress and biostimulant/nanoparticle treatments

Character	Control (0 mM)	Control (200 mM)	PGPEB	FeNPs	PGPEB + FeNPs	*p*-value
Root diameter (mm)	0.569 ± 0.005^a^	0.593 ± 0.008^b^	0.659 ± 0.008^b^^c^	0.577 ± 0.006^a^	0.675 ± 0.011^c^	< 0.05
Parenchyma thickness (mm)	0.130 ± 0.001^a^	0.138 ± 0.002^b^	0.157 ± 0.001^c^	0.119 ± 0.002^a^^b^	0.130 ± 0.001^a^	< 0.05
Number of parenchyma rows	6.06 ± 0.07^b^	5.97 ± 0.06^a^^b^	5.88 ± 0.09^a^	5.94 ± 0.11^a^	7.19 ± 0.07^c^	< 0.05
Distance between pith and vascular cylinder (mm)	0.100 ± 0.001^a^	0.130 ± 0.002^b^	0.182 ± 0.002^c^	0.139 ± 0.002^b^	0.139 ± 0.002^b^	< 0.05
Phloem thickness (mm)	0.021 ± 0.000^a^	0.033 ± 0.000^b^	0.035 ± 0.001^b^	0.030 ± 0.000^b^	0.038 ± 0.001^c^	< 0.05
Xylem vessel diameter (mm)	0.038 ± 0.001^b^	0.040 ± 0.000^b^	0.038 ± 0.001^b^	0.033 ± 0.000^a^	0.031 ± 0.000^a^	< 0.05
Xylem tissue thickness (mm)	0.061 ± 0.001^a^	0.084 ± 0.001^b^	0.082 ± 0.001^b^	0.085 ± 0.001^b^	0.083 ± 0.001^b^	< 0.05
Number of xylem vessels	7.07 ± 0.14^a^	9.02 ± 0.04^c^	8.07 ± 0.20^b^	6.94 ± 0.09^a^	7.90 ± 0.11^b^	< 0.05
Vascular cylinder width (mm)	0.047 ± 0.001^a^	0.053 ± 0.000^b^	0.050 ± 0.000^a^	0.042 ± 0.001^a^	0.058 ± 0.001^c^	< 0.05
Vascular cylinder thickness (mm)	0.110 ± 0.001^c^	0.099 ± 0.001^b^	0.110 ± 0.001^c^	0.093 ± 0.001^a^	0.105 ± 0.001^c^	< 0.05
Number of vascular cylinder rings	1.00 ± 0.00^a^	1.00 ± 0.00^a^	1.00 ± 0.00^a^	1.00 ± 0.00^a^	2.04 ± 0.03^b^	< 0.05
Number of pith parenchyma rows	6.00 ± 0.12^a^	10.00 ± 0.20^c^	11.00 ± 0.22^d^	9.00 ± 0.18^b^	9.00 ± 0.18^b^	< 0.05
Collenchyma thickness (mm)	0.025 ± 0.000^a^	0.031 ± 0.000^c^	0.031 ± 0.000^c^	0.033 ± 0.000^e^	0.030 ± 0.000^b^	< 0.05
Number of collenchyma rows	3.00 ± 0.00^a^	3.00 ± 0.00^a^	4.00 ± 0.00^a^	3.00 ± 0.00^a^	3.00 ± 0.00^a^	> 0.05
Number of sclerenchyma sheath rows around vascular cylinder	1.00 ± 0.01^b^	0.97 ± 0.02^a^	0.99 ± 0.01^a^	1.00 ± 0.01^b^	2.04 ± 0.03^c^	< 0.05

### Hierarchical clustering analysis of root anatomical traits

**Fig. 14:**
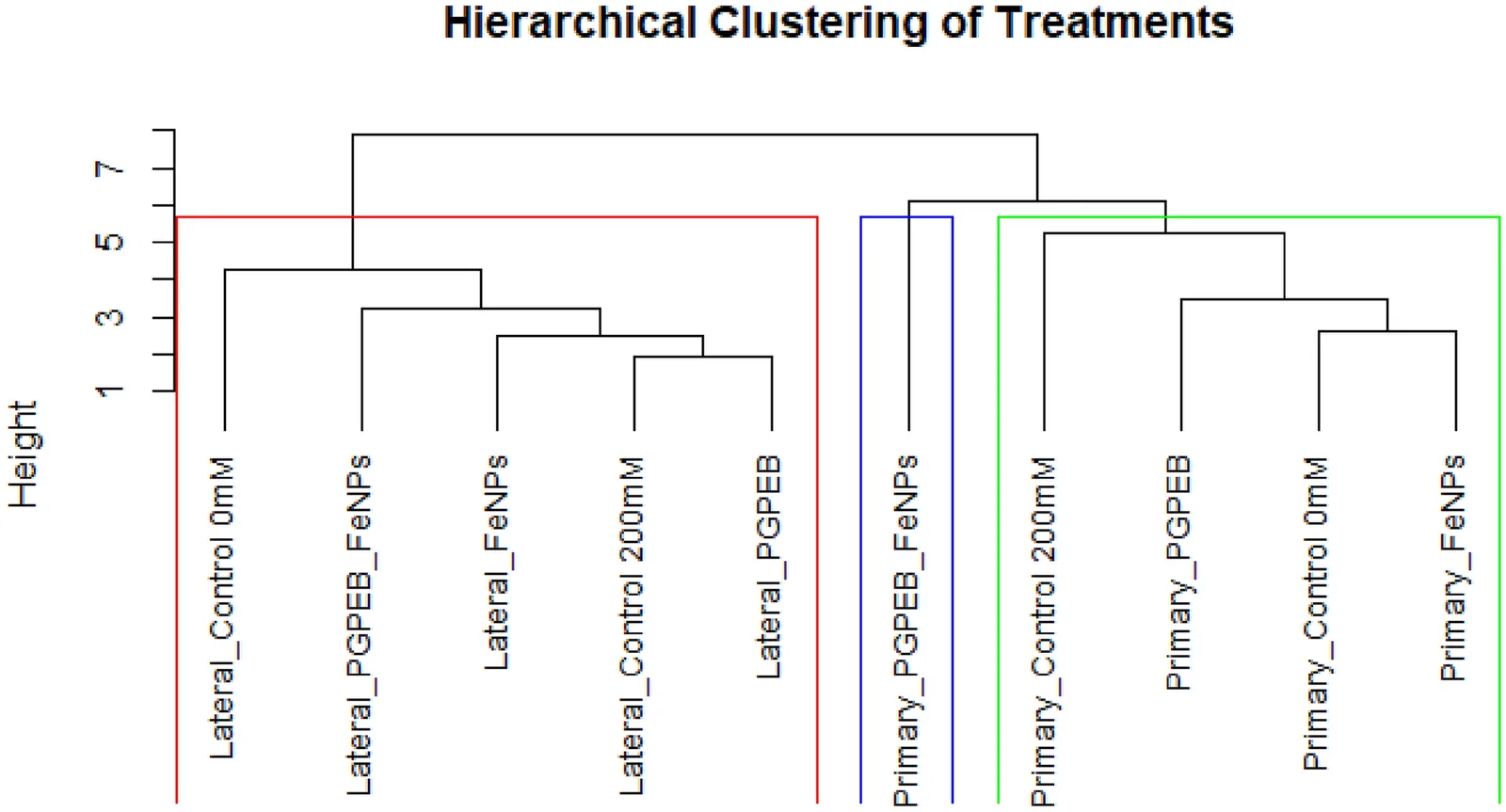
Hierarchical clustering analysis revealed clear grouping patterns among the different treatments and root types based on the evaluated anatomical traits. The dendrogram separated the samples into distinct clusters, indicating differential responses of primary and lateral roots to salinity stress and treatment applications

Primary root samples formed a relatively independent cluster from lateral root samples, demonstrating substantial anatomical variation between the two root systems under the tested conditions (Fig. 14). Within the primary root group, the PGPEB and FeNPs treatments showed closer similarity to the untreated control under non-saline conditions, suggesting that both treatments partially alleviated the adverse anatomical effects induced by salinity stress. In contrast, the salinity-stressed control (200 mM) displayed greater divergence, reflecting pronounced structural alterations caused by salt stress. For lateral roots, the FeNPs-loaded bacteria-treated samples clustered closely together, indicating comparable anatomical responses and potential 3.9protective effects against salinity-induced damage. The lateral root salinity control exhibited moderate separation from treated samples, further confirming the negative impact of salt stress on root structural organization. Overall, the clustering pattern suggests that FeNP-loaded PGPEB contributed to maintaining root anatomical integrity under salinity stress, while the combined treatment exhibited intermediate similarity between treated and control groups, indicating a potential synergistic influence on root structural adaptation.

### PCA-based discrimination of root anatomical characteristics among *Zea mays* treatments

**Fig. 15:**
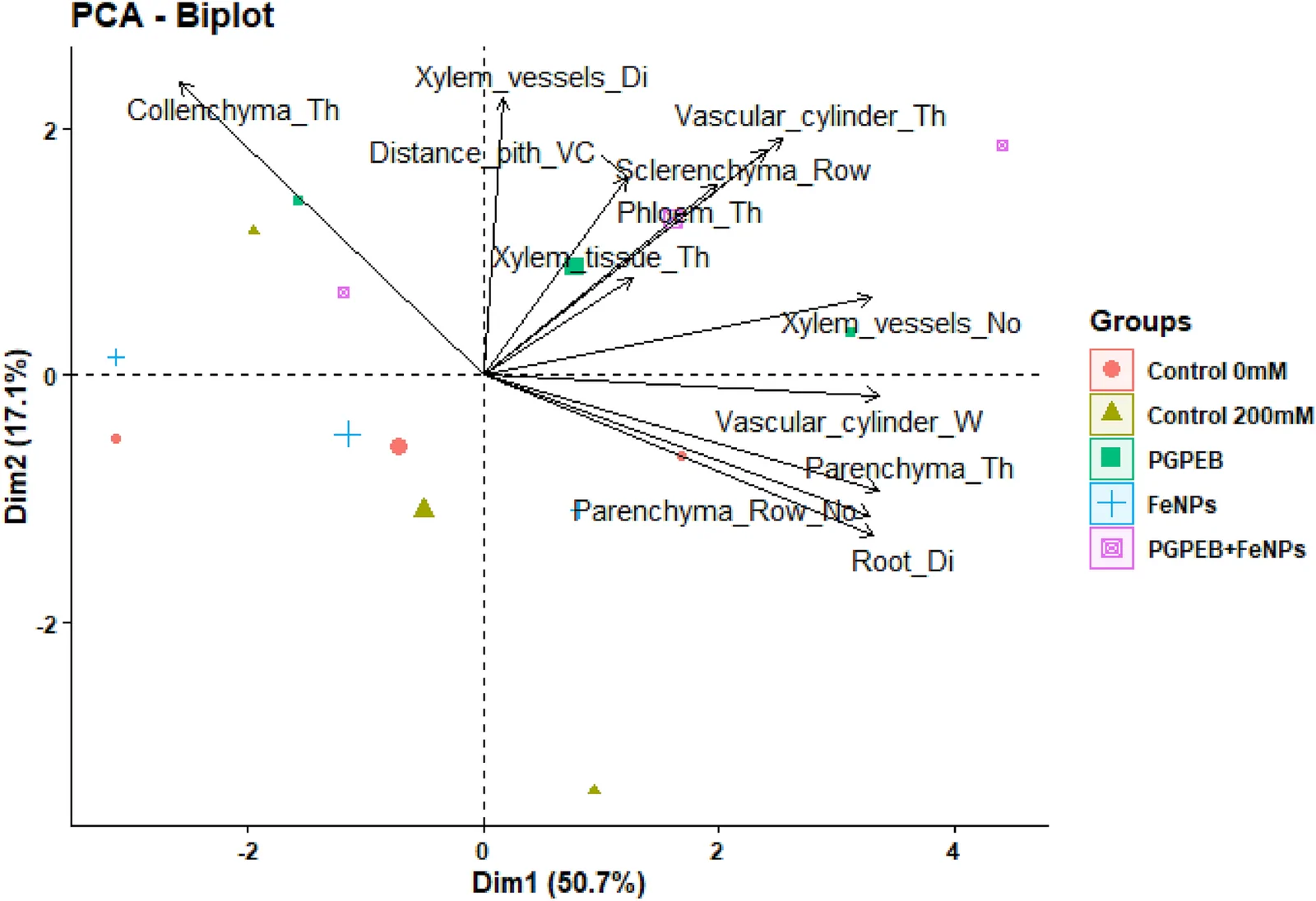
the principal component analysis (PCA) biplot illustrates a clear multivariate separation among *Zea mays* treatments based on root anatomical traits. The first two principal components explained a substantial proportion of the total variance (Dim1 = 50.7%, Dim2 = 17.1%), indicating that the selected anatomical parameters effectively capture the structural variation induced by salinity stress and biostimulant/nanoparticle applications

Along PC1 (Dim1), treatments such as PGPEB and FeNP-loaded PGPEB were strongly associated with positive loadings of key vascular traits (Fig. 15), including root diameter, vascular bundle width, xylem vessel number, and parenchyma-related parameters. This indicates enhanced overall root anatomical development and improved vascular differentiation under these treatments. In contrast, Control 200 mM (salinity stress) was positioned on the opposite side of the axis, reflecting its association with reduced structural development and stress-related anatomical constraints. On PC2 (Dim2), separation was mainly driven by traits such as collenchyma thickness, distance pith–vascular bundle, and xylem vessel diameter, suggesting that these features contribute to secondary structural differentiation and mechanical reinforcement responses under stress conditions. Overall, the PCA clearly demonstrates that PGPEB and FeNP-loaded PGPEB treatments promote a coordinated enhancement of vascular and supportive tissues, while salinity stress alone leads to a distinct reduction in anatomical complexity. The clear clustering pattern confirms strong treatment-dependent modulation of root structural traits in *Zea mays*.

## Discussion

Salinity stress severely affects plant growth and productivity through osmotic imbalance ion toxicity, oxidative stress, and structural cellular damage, particularly in glycophytic crops such as maize [62]. The growth curve analysis demonstrated that the isolate exhibited a typical bacterial growth pattern with a distinct exponential phase and sustained viability before entering the stationary phase. Harvesting bacterial cells during the late exponential phase ensured metabolically active cells for FeNPs immobilization, which may have contributed to efficient root colonization and improved plant growth-promoting performance under salinity stress.

The close association between FeNPs and bacterial surfaces is likely mediated by electrostatic interactions and the binding of nanoparticle surfaces to negatively charged functional groups, including carboxyl, phosphate and hydroxyl groups present in the bacterial cell envelope and extracellular polymeric substances. These interactions facilitate nanoparticle attachment while maintaining bacterial morphology. In the present study, severe salinity stress (200 mM NaCl) induced substantial physiological, biochemical, ultrastructural, and anatomical alterations in *Zea mays* seedlings. However, treatments with plant growth-promoting endophytic bacteria (PGPEB), iron nanoparticles (FeNPs), and particularly FeNP-loaded PGPEB significantly alleviated these adverse effects, indicating that the integration of microbial and nanotechnological approaches provides an effective strategy for enhancing plant tolerance under saline conditions. The superior performance of the FeNPs-loaded PGPEB treatment suggests that the combined application of PGPEB and FeNPs provided greater protection against salinity than either treatment alone. Although formal synergy analysis was not performed, the observed responses indicate enhanced beneficial effects resulting from the combined nano–bio treatment. Transmission electron microscopy (TEM) confirmed the successful adsorption of FeNPs onto the surface of the endophytic bacterial isolate recovered from *Zea mays* roots. The preservation of bacterial morphology, intact cell envelopes, and flagellar structures after nanoparticle loading indicated that the immobilization process did not cause detectable structural damage to the bacterial cells, suggesting that the bacterial carrier remained structurally intact. This observation is particularly important because maintaining bacterial structural integrity is essential for effective root colonization and the expression of plant growth-promoting traits. The TEM analysis revealed a primary particle size ranging from 16.7 to 54.7 nm, which falls within the effective nanoscale range previously reported for enhanced nutrient delivery and biological activity in plant systems [63]. The complementary physicochemical characterization further supported the suitability of the commercially obtained FeNPs for biological application. X-ray diffraction (XRD) analysis confirmed the crystalline iron oxide phase, while Fourier Transform Infrared Spectroscopy (FTIR) verified the characteristic Fe–O bonds and surface functional groups. Dynamic light scattering (DLS) revealed a larger hydrodynamic particle size than that observed by TEM, which is expected because DLS measures nanoparticles in their hydrated state together with the surrounding solvation layer and limited aggregation in suspension. In addition, the measured negative zeta potential indicated moderate colloidal stability, suggesting sufficient electrostatic repulsion to minimize excessive aggregation during immobilization. Collectively, these complementary analyses confirmed the morphology, crystallinity, surface chemistry, particle size characteristics, and colloidal behavior of the FeNPs, supporting their suitability for developing the FeNPs-immobilized PGPEB formulation. Owing to their high surface-area-to-volume ratio, nanoparticles within this size range exhibit enhanced reactivity and bioavailability, potentially facilitating interactions with both microbial cells and plant tissues.

The close attachment of FeNPs to bacterial surfaces suggests the formation of a nano-bio hybrid system in which bacterial cells may serve as biological carriers for nanoparticle delivery. The complementary physicochemical characterization supports the suitability of the FeNPs for immobilization onto PGPEB and subsequent application under salinity stress. Microbial-assisted nanoparticle transport may improve iron availability in the rhizosphere and within plant tissues while simultaneously reducing nanoparticle aggregation and enhancing their persistence under stress conditions [64]. In addition, increased iron availability may support bacterial metabolic processes involved in respiration, antioxidant defense, and synthesis of plant growth-promoting metabolites. Consequently, the bacterial cell functions not only as a plant growth-promoting agent but also as an effective biological carrier for targeted micronutrient delivery, a concept that has gained increasing attention in nano-enabled microbial biotechnology [64]. The beneficial effects of the FeNP-loaded PGPEB treatment were evident during seed germination and seedling establishment under saline conditions. Salinity is known to inhibit germination through osmotic constraints, ionic toxicity, and metabolic disturbances that impair water uptake and enzyme activation [65]. The improved germination observed following application of the nano-bio formulation may be attributed to enhanced microbial survival, improved nutrient availability, and more efficient modulation of stress-responsive pathways. Endophytic bacteria are capable of producing phytohormones, solubilizing nutrients, and regulating plant stress signaling [66, 67], while nanoparticle-mediated enhancement of microbial performance may further strengthen these functions. The stronger response observed under high salinity levels supports the hypothesis that nano-enabled microbial inoculants become increasingly effective as environmental stress intensifies, consistent with previous reports on nanotechnology-assisted beneficial microorganisms [68]. Similarly, improvements in shoot growth under saline conditions likely resulted from the combined action of bacterial metabolism and enhanced iron nutrition. Iron is a critical component of photosynthetic electron transport systems and numerous enzymatic pathways [69, 70]. Unlike previous studies that evaluated PGPEB or FeNPs separately, the present work demonstrates that loading FeNPs onto PGPEB was associated with greater improvements in germination and early seedling establishment under severe salinity, supporting the potential of this nano–bio formulation for stress mitigation.

FeNPs application may have improved iron availability, thereby supporting photosynthetic electron transport and enzymatic processes, although iron accumulation was not directly quantified in the present study. through FeNP delivery may therefore contribute to improved photosynthetic performance and metabolic activity. Simultaneously, endophytic bacteria may stimulate the production of growth-promoting compounds such as indole−3-acetic acid (IAA), thereby enhancing cell elongation and shoot development. The significant interaction between treatment and salinity suggests that these mechanisms become particularly important under severe stress conditions, where nutrient imbalance and oxidative damage strongly constrain plant growth. The biochemical responses further support the existence of coordinated nano-bio-mediated stress mitigation mechanisms. Salinity-induced accumulation of reactive oxygen species (ROS) disrupts cellular homeostasis and causes oxidative damage to membranes, proteins, and nucleic acids [71, 72]. The enhanced activities of SOD, CAT, and POD in treated plants indicate activation of an efficient antioxidant defense network capable of limiting ROS accumulation. Iron serves as an essential cofactor for numerous redox-active enzymes, whereas endophytic bacteria can induce systemic stress responses and modulate antioxidant metabolism [73]. The combination of these effects likely explains the substantial reduction in lipid peroxidation observed in the FeNP-loaded PGPEB treatment, as reflected by lower MDA levels. Furthermore, enhanced proline accumulation and improved relative water content suggest more effective osmotic adjustment and maintenance of cellular hydration under saline conditions.

Hormonal regulation also appears to contribute to the protective effects of the nano-bio formulation. Increased IAA concentrations in treated plants indicate stimulation of growth-promoting pathways, whereas the lower ABA levels compared with salt-stressed controls suggest reduced stress perception and improved physiological adaptation. Such hormonal modulation is consistent with previous studies demonstrating the ability of endophytic microorganisms to influence plant hormone homeostasis under abiotic stress conditions [74]. The ultrastructural observations provide direct evidence for the protective role of the nano-bio hybrid system. Severe plasmolysis, membrane detachment, and cytoplasmic shrinkage observed in salt-stressed roots are typical manifestations of osmotic and oxidative injury [75, 76]. In contrast, roots treated with PGPEB and particularly FeNP-loaded PGPEB maintained membrane-cell wall continuity and preserved cytoplasmic organization. These findings suggest that the combined treatment improved membrane stability and cellular resilience against salt-induced dehydration. Enhanced antioxidant activity, improved nutrient acquisition, and more efficient osmotic regulation likely contributed collectively to the preservation of cellular architecture [77, 78].

The heterogeneous distribution of bacterial cells within cortical and vascular tissues indicates active endophytic colonization and highlights the dynamic nature of plant–microbe interactions under stress conditions [79]. Such colonization patterns may facilitate localized delivery of nutrients, signaling molecules, and protective metabolites throughout the root system. This spatial association between microbial cells and plant tissues may further enhance the effectiveness of nanoparticle transport and utilization within the host plant. The anatomical improvements observed in both primary and lateral roots further support the role of the nano-bio formulation in maintaining root functionality under salinity stress. Salinity-induced reductions in xylem vessel dimensions, phloem thickness, and overall vascular organization can severely impair water and nutrient transport [80, 81]. The restoration of these anatomical characteristics following treatment with PGPEB and FeNPs indicates improved tissue differentiation and structural stability. Notably, the FeNP-loaded PGPEB treatment produced the greatest enhancement in vascular development and supportive tissues, suggesting improved conductive capacity and mechanical reinforcement. These structural modifications likely contribute to the improved physiological performance observed under severe salinity stress [82]. Collectively, the present findings demonstrate that the combined application of PGPEB and FeNPs effectively improved maize tolerance to salinity by enhancing antioxidant defense, osmotic adjustment, root anatomical organization, and cellular ultrastructure. The TEM observations further indicated successful association of FeNPs with bacterial cells and their localization within maize root tissues, providing additional insight into nano–bio interactions under saline conditions. Although the precise mechanisms underlying these interactions require further investigation, particularly with respect to iron uptake and bacterial persistence, the present study highlights the potential of FeNP-loaded PGPEB as an environmentally sustainable strategy for improving crop performance under saline conditions.

## Data Availability

No datasets were generated or analysed during the current study.

## References

[1] Singh K, Saha N, Borah AS, Borah PP, Khanikar L, Garai S, et al. The hidden enemy: how salinity rewires plant defenses and what we can do about it. Plant Stress. 2025;17:100990.

[2] Majeed A, Muhammad Z. Salinity: a major agricultural problem—causes, impacts on crop productivity and management strategies. In: Hasanuzzaman M, Hakeem K, Nahar K, Alharby H, editors. Plant abiotic stress tolerance: agronomic, molecular and biotechnological approaches. Cham: Springer; 2019. p. 83–99.

[3] FAO. Global status of salt-affected soils: main report. Rome: Food and Agriculture Organization of the United Nations; 2024.

[4] Huang J, Grover A. Salinity stress in agriculture: causes, impacts and recent advances in mitigation. Plant Soil. 2025;508:1–25.

[5] Chen J, Liu Y, Wang H, Zhang X, Li M, Sun Q, et al. Recent advances in understanding salinity tolerance mechanisms in cereal crops. Crop J. 2025;13(2):245–62.

[6] Mohanavelu A, Naganna SR, Al-Ansari N. Irrigation-induced salinity and sodicity hazards on soil and groundwater: an overview of its causes, impacts and mitigation strategies. Agriculture. 2021;11(10):983.

[7] Arif Y, Singh P, Siddiqui H, Bajguz A, Hayat S. Salinity-induced physiological and biochemical changes in plants: an omic approach towards salt stress tolerance. Plant Physiol Biochem. 2020;156:64–77. 32906023 10.1016/j.plaphy.2020.08.042

[8] Dell’Aversana E, Hessini K, Ferchichi S, Fusco GM, Woodrow P, Ciarmiello LF, et al. Salinity duration differently modulates physiological parameters and metabolite profiles in roots of two contrasting barley genotypes. Plants. 2021;10(2):307. 33562862 10.3390/plants10020307 PMC7914899

[9] Gupta A, Bano A, Rai S, Mishra R, Singh M, Sharma S, et al. Mechanistic insights of plant–microbe interaction towards drought and salinity stress in plants for enhancing agriculture productivity. Plant Stress. 2023;10:100184.

[10] Sarkar AK, Sadhukhan S. Impact of salinity on growth and development of plants with central focus on glycophytes: an overview. Bull Env Pharmacol Life Sci. 2023;12:235–66.

[11] Hasanuzzaman M, Raihan MRH, Masud AAC, Rahman K, Nowroz F, Rahman M, et al. Regulation of reactive oxygen species and antioxidant defense in plants under salinity. Int J Mol Sci. 2021;22(17):9326. 34502233 10.3390/ijms22179326 PMC8430727

[12] Ahmed B, Khan A, Khan MS, Lee J. Mitigation of salinity stress in plants by plant growth-promoting microbes and their metabolites. Microbiol Res. 2023;272:127389.37099956

[13] Ansabayeva A, Yermagambetova M, Akhmetova M, Toleugali A, Bekkuzhina S, Beibitova A, et al. Plant growth-promoting microbes for resilient farming systems: mitigating environmental stressors and boosting crop productivity—a review. Horticulturae. 2025;11(3):260.

[14] Alhammad BA, Ahmad A, Seleiman MF. Nano-hydroxyapatite and ZnO-NPs mitigate Pb stress in maize. Agronomy. 2023;13(4):1174.

[15] El-Naggar AH, Al-Sabagh AM. Physiological and anatomical adaptations of maize (Zea mays L.) under saline irrigation. J Plant Physiol. 2025;299:154258.

[16] Zhang H, Liu Y, Chen X, Wang J, Li S, Zhao P, et al. Salinity-induced morphological, anatomical and ultrastructural alterations in maize (Zea mays L.) and their amelioration by exogenous treatments. Environ Exp Bot. 2023;213:105420.

[17] Li X, Wang Y. Glycophyte responses to salinity stress: mechanisms and improvement strategies. Plant Cell Environ. 2025;48(4):901–18.

[18] Maas EV, Hoffman GJ. Crop salt tolerance—current assessment. J Irrig Drain Div. 1977;103(2):115–34.

[19] Islam M, Sandhi A, Hossain MR, Chowdhury AT, Munira S. Salinity stress in maize: consequences, tolerance mechanisms, and management strategies. OBM Genet. 2024;8(2):231.

[20] Kumar P, Choudhary M, Halder T, Prakash NR, Singh V, Vinothkumar B, et al. Deciphering seedling-stage salinity stress tolerance in maize genotypes through morpho-physiological and ionic traits. Plants. 2025;14(7):1037. 41977226 10.3390/ijms27073037 PMC13073411

[21] Li N, Zhao Y, Wang Q, Sun H, Zhou X, Liu D. Salt-induced leaf anatomical and mesophyll structural changes in cereals: implications for photosynthesis. Photosynth Res. 2025;163(2):199–214.

[22] Rajput VD, Minkina T, Sushkova S, Mandzhieva S, Tsitsuashvili V, Ranjan A, et al. Structural and ultrastructural changes in nanoparticle exposed plants. In: Panpatte D, Jhala Y, editors., et al., Nanoscience for sustainable agriculture. Cham: Springer; 2019. p. 281–95.

[23] Elemike EE, Uzoh IM, Onwudiwe DC, Babalola OO. The role of nanotechnology in the fortification of plant nutrients and improvement of crop production. Appl Sci. 2019;9(3):499.

[24] Naskar S, Palit PK. Anatomical and physiological adaptations of mangroves. Wetl Ecol Manag. 2015;23(3):357–70.

[25] Santoyo G, Moreno-Hagelsieb G, del Carmen Orozco-Mosqueda M, Glick BR. Plant growth-promoting bacterial endophytes. Microbiol Res. 2016;183:92–9. 26805622 10.1016/j.micres.2015.11.008

[26] Egamberdieva D, Wirth SJ, Alqarawi AA, AbdAllah EF, Hashem A. Phytohormones and beneficial microbes: essential components for plants to balance stress and fitness. Front Microbiol. 2017;8:2104. 29163398 10.3389/fmicb.2017.02104 PMC5671593

[27] Sherzad Z, Nawakht NA, Sherzad F. Plant growth-promoting endophytic bacteria: a sustainable solution for climate change and environmental stresses in agriculture. Discov Appl Sci. 2025;7(8):894.

[28] Gupta S, Grover A. Plant growth-promoting rhizobacteria and engineered nanomaterials for sustainable mitigation of abiotic stress. Plant Stress. 2023;9:100174.

[29] Yousaf MJ, Hussain A, Hamayun M, Iqbal A, Irshad M, Khan SA, et al. Iron and zinc micronutrients and plant abiotic stress tolerance. Plant Stress. 2023;9:100179.

[30] Asif S, Jan R, Kim N, Asaf S, Lubna, Khan MA, et al. Halotolerant endophytic bacteria alleviate salinity stress in rice (*Oryza**sativa* L.) by modulating ion content, endogenous hormones, the antioxidant system and gene expression. BMC Plant Biol. 2023;23:494. 10.1186/s12870-023-04517-z PMC10576267 37833628

[31] Liang Q, Tan D, Chen H, Guo X, Afridi MS, Wang X, et al. Endophyte-mediated enhancement of salt resistance in *Arachis**hypogaea* L by regulation of osmotic stress and plant defense-related genes. Front Microbiol. 2024;15:1383545. 38846577 10.3389/fmicb.2024.1383545 PMC11153688

[32] Nath R, Moulick BCD, Hossain A. Green nanofertilizers for sustainable agriculture. In: Green nanotechnology for stress-resilient crops and sustainable agroecosystems. Cham: Springer; 2026.

[33] Naseem J, Iqbal S, Saddiq MS, Hussain S, Khaliq A, Ramzan M, et al. Green-synthesized FeNPs ameliorate drought stress in *Spinacia oleracea* L. through improved photosynthetic capacity, redox balance, and antioxidant defense. Sci Rep. 2025;15(1):1782. 10.1038/s41598-024-84061-4 PMC11729898 39805888

[34] Mukhtiar A, Zia MA, Alawadi HFN, Naqve M, Seleiman MF, Mahmood A, et al. Role of iron oxide nanoparticles in maize (*Zea mays* L.) to enhance salinity stress tolerance. Not Bot Horti Agrobot Cluj-Napoca. 2024;52(3):13695.

[35] Kreslavski VD, Shmarev AN, Ivanov AA, Zharmukhamedov SK, Strokina V, Kosobryukhov A, et al. Effects of iron oxide nanoparticles (Fe₃O₄) and salinity on growth, photosynthesis, antioxidant activity and distribution of mineral elements in wheat (*Triticum aestivum*). Funct Plant Biol. 2023;50(11):932–40. 37573788 10.1071/FP23085

[36] Alharbi K, Hafez E, Omara AE-D, Awadalla A, Nehela Y. Plant growth-promoting rhizobacteria and silica nanoparticles stimulate sugar beet resilience to irrigation with saline water in salt-affected soils. Plants. 2022;11(22):3117. 36432846 10.3390/plants11223117 PMC9694940

[37] Sun Y, Ma L, Ma J, Li B, Zhu Y, Chen F. Combined application of plant growth-promoting bacteria and iron oxide nanoparticles ameliorates the toxic effects of arsenic in ajwain (*Trachyspermum ammi* L.). Front Plant Sci. 2022;13:1098755. 10.3389/fpls.2022.1098755 PMC9832315 36643291

[38] Aisida SO, Köse A, Koçer AT, Bingöl AB, Oktay B, Karaduman E, et al. Recent advances in synthesis and functionalization of iron oxide nanoparticles as a promising material toward precision biomedical applications. Biomed Mater Devices. 2026;1–28.

[39] Caf M, Esmaeilnejad-Ahranjani P, Kolosnjaj-Tabi J, Sabotic J, Berlec A, Zaversek N, et al. Magnetic field-driven strategies for biofilm disruption: From iron oxide nanoparticles to adaptive swarms of magnetic microrobots. ACS Nano. 2026;20(1):34–58. 41477707 10.1021/acsnano.5c14390 PMC12810490

[40] Nadeem F, Ahmad Z, Wang R, Han J, Shen Q, Chang F, et al. Foxtail millet (*Setaria italica* (L.) Beauv.) grown under low nitrogen shows a smaller root system, enhanced biomass accumulation, and nitrate transporter expression. *Front Plant Sci*. 2018;9:205. 10.3389/fpls.2018.00205 PMC5826958 29520286

[41] Sahu PK, Tilgam J, Mishra S, Hamid S, Gupta A, Verma SK, et al. Surface sterilization for isolation of endophytes: ensuring what (not) to grow. J Basic Microbiol. 2022;62(6):647–68. 35020220 10.1002/jobm.202100462

[42] Ramalashmi K, Prasanna VK, Magesh K, Sanjana R, Siril JS, Ravibalan K. A potential surface sterilization technique and culture media for the isolation of endophytic bacteria from *Acalypha indica* and its antibacterial activity. J Med Plants Stud. 2018;6(1):181–4.

[43] Ryan RP, Germaine K, Franks A, Ryan DJ, Dowling DN. Bacterial endophytes: recent developments and applications. FEMS Microbiol Lett. 2008;278(1):1–9. 18034833 10.1111/j.1574-6968.2007.00918.x

[44] Monteiro RA, Balsanelli E, Wassem R, Marin AM, Brusamarello-Santos LCC, Schmidt MA, et al. Sugarcane growth promotion by the endophytic bacterium *Pantoea agglomerans* 33.1. Appl Environ Microbiol. 2012;78(21):7511–22. 10.1128/AEM.00836-12 PMC3485700 22865062

[45] Lane DJ. 16S/23S rRNA sequencing. In: Stackebrandt E, Goodfellow M, editors. Nucleic acid techniques in bacterial systematics. New York: Wiley; 1991. p. 115–75.

[46] Selim S, Negrel J, Govaerts C, Gianinazzi S, van Tuinen D. Isolation and partial characterization of antagonistic peptides produced by *Paenibacillus* sp. strain B2 isolated from the sorghum mycorrhizosphere. Appl Environ Microbiol. 2005;71(10):6501–7. 10.1128/AEM.71.11.6501-6507.2005 PMC1287738 16269674

[47] Zhu H, Han J, Xiao JQ, Jin Y. Uptake, translocation and physiological effects of magnetic iron oxide (γ-Fe₂O₃) nanoparticles in corn (*Zea mays* L.). Chemosphere. 2016;159:326–34. 10.1016/j.chemosphere.2016.05.083 27314633

[48] Bozzola JJ, Russell LD. Electron microscopy: principles and techniques for biologists. 2nd ed. Sudbury (MA): Jones and Bartlett Publishers; 1999.

[49] Schneider CA, Rasband WS, Eliceiri KW. NIH Image to ImageJ: 25 years of image analysis. Nat Methods. 2012;9(7):671–5. 22930834 10.1038/nmeth.2089 PMC5554542

[50] Gul N, Ahmad N, Kanwal Z, Rizvi SA, Khan F, Bibi M. Green synthesis of iron nanoparticles: structural characterisation and advanced environmental applications. Spectrum Eng Sci. 2026;4(6):3661–79.

[51] Zhang X, Wen J, Ba Y, Gong Z. Novel green iron nanoparticle embedded hydrogel for removal of heavy metals and dyes: synergistic removal and diverse pathways. J Water Process Eng. 2026;89:110308.

[52] Ali A, Abbas M, Sarfraz K, Hussain A, Khan F. Plant-mediated green synthesis of iron nanoparticles for advanced environmental remediation: Mechanistic design, characterization, applications, and sustainability outlook. Spectrum Eng Sci. 2026;4(6):3236–54.

[53] Ranmadugala D, Ebrahiminezhad A, Manley-Harris M, Ghasemi Y, Berenjian A. Magnetic immobilization of bacteria using iron oxide nanoparticles. Biotechnol Lett. 2018;40(2):237–50. 29181762 10.1007/s10529-017-2477-0

[54] Barrs HD, Weatherley PE. A re-examination of the relative turgidity technique for estimating water deficits in leaves. Aust J Biol Sci. 1962;15(3):413–28.

[55] Heath RL, Packer L. Photoperoxidation in isolated chloroplasts: I. Kinetics and stoichiometry of fatty acid peroxidation. Arch Biochem Biophys. 1968;125(1):189–98. 10.1016/0003-9861(68)90654-1 5655425

[56] Bates LS, Waldren RP, Teare ID. Rapid determination of free proline for water-stress studies. Plant Soil. 1973;39(1):205–7.

[57] Beauchamp C, Fridovich I. Superoxide dismutase: improved assays and an assay applicable to acrylamide gels. Anal Biochem. 1971;44(1):276–87. 4943714 10.1016/0003-2697(71)90370-8

[58] Chance B, Maehly AC. Assay of catalases and peroxidases. Methods Enzymol. 1955;2:764–75. 10.1002/9780470110171.ch14 13193536

[59] Nakurte I, Keisa A, Rostoks N. Development and validation of a reversed-phase liquid chromatography method for the simultaneous determination of indole-3-acetic acid, indole-3-pyruvic acid, and abscisic acid in barley (*Hordeum vulgare* L.). J Anal Methods Chem. 2012;2012:103575. 10.1155/2012/103575 PMC3335325 22567549

[60] Ruzin SE. Plant microtechnique and microscopy. New York: Oxford University Press; 1999.

[61] R Core Team. *R: *a language and environment for statistical computing. Vienna: R Foundation for Statistical Computing; 2024. Available from: https://www.R-project.org/.

[62] Kataria S, Verma SK. Salinity stress responses and adaptive mechanisms in major glycophytic crops: the story so far. In: Salinity responses and tolerance in plants, volume 1: targeting sensory, transport and signaling mechanisms. Cham: Springer; 2018. p. 1–39.

[63] Vishwakarma K, Kumar N, Lateef A. Microbiome and nano-cross-talk: sustainable agriculture and beyond. Elsevier; 2024.

[64] Theresa M, et al. Agricultural sustainability through nanotechnology. CRC Press; 2025.

[65] Uçarlı C. Effects of salinity on seed germination and early seedling stage. In: Abiotic stress in plants. IntechOpen; 2020.

[66] Gari TH. Seed germination and early seedling growth response of selected sorghum (*Sorghum bicolor* L. Moench) under varying salinity condition. Haramaya University; 2023.

[67] Mohammed SP, Jakkula D, Srinivasan R. Recent advances in endophyte-mediated biotic and abiotic stress tolerance in plants. Planta. 2026;263(5):130. 41963631 10.1007/s00425-026-04996-y

[68] Upadhyay SK, et al. Mechanisms and applicability of nanotechnology-mediated beneficial microbes in mitigation of salinity stress in plants. Plant Physiol Biochem. 2025:110306. 10.1016/j.plaphy.2025.110306 40749465

[69] Mbarki S, et al. Strategies to mitigate the salt stress effects on photosynthetic apparatus and productivity of crop plants. In: Salinity responses and tolerance in plants, volume 1. Springer; 2018. p. 85–136.

[70] Yousaf N, et al. Characterization of root and foliar-applied iron oxide nanoparticles in improving maize (*Zea mays* L.) performance. Nanomaterials. 2023;13(23):3036. 10.3390/nano13233036 PMC10708543 38063732

[71] Lau SE, et al. Enhancing plant resilience to abiotic stress: the power of biostimulants. Phyton. 2025;94(1):1.

[72] Gill SS, et al. Generation and scavenging of reactive oxygen species in plants under stress. In: Improving crop resistance to abiotic stress. 2012. p. 49–70.

[73] Yasmeen F, et al. Current scenario and future trends of plant nano-interaction to mitigate abiotic stresses: a review. In: Impact of engineered nanomaterials in genomics and epigenomics. 2023. p. 355–96.

[74] Alqudah AM, Safhi FA, Thabet SG. Genetic insights into the synergistic effects of nano-iron on yield, quality, and antioxidant defense in barley under salt stress. Mol Genet Genomics. 2025;300:54. 40459609 10.1007/s00438-025-02254-6

[75] Bilska A, Suski S, Sowinski P. Electron tomography technique–useful tool for plasmodesmata ultrastructure analysis in maize leaves. BioTechnologia. 2013;94(3).

[76] Tufail MA, et al. Can bacterial endophytes be used as a promising bio-inoculant for the mitigation of salinity stress in crop plants?—a global meta-analysis (2011–2020). Microorganisms. 2021;9(9):1861. 34576756 10.3390/microorganisms9091861 PMC8467090

[77] Yousaf MA, et al. Micronutrient nanoprotectants curtail arsenic-induced physio-oxidative damage. Environ Sci Nano. 2026;13(1):582–603.

[78] Kaur R, et al. From microscopy to omics: tools and techniques in studying endophytic adaptation. J Plant Growth Regul. 2025;44(12):6766–84.

[79] Sabagh AE, et al. Salinity stress in cotton: adverse effects, survival mechanisms and management strategies. In: Engineering tolerance in crop plants against abiotic stress. CRC Press; 2021. p. 59–80.

[80] Zhao N, et al. Advanced imaging-enabled understanding of cell wall remodeling mechanisms mediating plant drought stress tolerance. Front Plant Sci. 2025;16:1635078. 40860734 10.3389/fpls.2025.1635078 PMC12371428

[81] Orozco-Mosqueda MDC, Glick BR, Santoyo G. Cross-talk within plant niches: endophytic and arbuscular mycorrhizal fungi for sustainable crop production. FEMS Microbiol Rev. 2026;50:fuaf063. 41388904 10.1093/femsre/fuaf063 PMC12766460

[82] Tripathi R, Singh D, Imam Z, Singh VK, Parveen R, Singh A, et al. Genetic architecture and breeding strategies for enhancing wheat (*Triticum aestivum* L.) tolerance to combined heat, drought, and salinity stresses. Mol Biol Rep. 2026;53(1):437.

